# Ethnoveterinary medicinal plants and their utilization by indigenous and local communities of Dugda District, Central Rift Valley, Ethiopia

**DOI:** 10.1186/s13002-024-00665-0

**Published:** 2024-03-09

**Authors:** Bula Kere Oda, Ermias Lulekal, Bikila Warkineh, Zemede Asfaw, Asfaw Debella

**Affiliations:** 1 Department of Plant Biology and Biodiversity Management, College of Natural and Computational Sciences, Addis Ababa University, P.O. Box 1176, Addis Ababa, Ethiopia; 2 Department of Biology, College of Natural and Computational Sciences, Bule Hora University, P.O. Box 144, Bule Hora, Ethiopia; 3 Traditional and Modern Medicine Research Directorate, Traditional Medicine, Ethiopian Public Health Institute, P.O. Box 1242/5654, Addis Ababa, Ethiopia

**Keywords:** Dugda District, Ethiopia, Ethnoveterinary uses, Medicinal plants, Livestock diseases

## Abstract

**Background:**

Ethnoveterinary medicinal plants have been used by the people of Dugda District in the primary health care system to treat various livestock ailments for generations, particularly, in underserved rural areas. However, these ethnoveterinary practices and medicinal plants are dwindling without proper documentation, due to undergoing socio-cultural and environmental changes. Hence, this study aimed at inventory and analysis of ethnoveterinary medicinal plants and the associated indigenous and local knowledge used in the treatment of livestock health problems in Dugda District.

**Methods:**

Data were collected from 378 local inhabitants through semi-structured interviews, 18 focus group discussions with 6 to 8 participants in each couple with participant field observations. Informant consensus factor (ICF), fidelity-level (FL) and relative importance value (RI) were used to evaluate the agreement of informants on ethnoveterinary practices, healing potential of medicinal plants and the most multipurpose species. Using standard taxonomic procedures, voucher specimens were collected, identified and deposited at the National Herbarium of Addis Ababa University.

**Results:**

In total, 64 medicinal plants of ethnoveterinary uses, belonging to 33 families were reported to treat 37 livestock health problems. Anthrax, inappetence and diarrhoea were said to be the most prevalent veterinary health problems treated by traditional medicinal plants. Family Fabaceae was the most widely reported (7 spp.), followed by Apocynaceae, Cucurbitaceae and Solanaceae (5 spp. each). Herbs were the dominant life forms (21spp.), followed by shrubs (20 spp.). The most commonly sought plant parts were leaves (55.25%), followed by roots (23.44%). The principal method of preparation was pounding remedial parts (46.85%) and mixing with cold water. The main route of administration was via oral application (72.67%), drenching diseased livestock. *Withania somnifera* and *Kedrostis foetidissima* were the most cited medicinal plants with 53 and 43 use reports (URs), respectively*.* ICF showed that respiratory diseases scored the highest value (0.94), while most of the reported medicinal plants were gastrointestinal agents. RI value analysis revealed that *Croton macrostachyus* had highest diversity of uses, followed by *K. foetidissima* and *W. somnifera*. Ethnoveterinary uses of some medicinal plants such as *Phytolacca dodecandra*, *Calpurnia aurea*, *Cyphostemma cyphopetalum* and *Verbascum sinaiticum* as prophylaxis against rabies were a new input for ethnoveterinary database.

**Conclusion:**

The study revealed that the people of the Dugda District are endowed with rich ethnoveterinary knowledge and medicinal plants. However, ethnoveterinary knowledge associated with medicinal plant species significantly differ (*P* < 0.05) between general and key informants, young and matured age groups and informants’ educational level. This infers the impact of mode of ethnoveterinary knowledge transfer, literacy, modern education and globalization on ethnoveterinary uses of medicinal plant species. And also most of the medicinal plants are found in wild habitats with nearly no conservation attention. Therefore, it is imperative to implement participatory conservation actions for medicinal plants in collaboration with traditional institutions (*Gada* system). The disparity of ethnoveterinary knowledge could be also minimized through awareness creation among local communities about the knowledge and its revitalization. Furthermore, medicinal plants, which are widely utilized and multipurpose, should be screened for their phytochemicals, pharmacological and toxicological activities to confirm ethnoveterinary uses and for future development of veterinary pharmaceuticals.

## Introduction

Ethnoveterinary medicine is a complex system of beliefs, knowledge, skills and practices concerning animal husbandry as well as general animal care [1]. Although it mainly focuses on the use of ethnoveterinary medicinal plants to treat animal diseases, the practices of ethnoveterinary medicine also include the use of diagnostic procedures, animal husbandry practices and surgical methods [2]. Recently, this system has gained attention for its potential usefulness in contributing to livestock well-being, particularly at the level of primary animal healthcare [3]. In many rural areas of the world, particularly in remote and marginal areas, traditional herbal medicines are crucial to treating domestic animals. This is due to several reasons, including cost-effectiveness, accessibility, efficacy and safety; one remedy for various ailments; and viable alternatives to allopathic drugs [4, 5]. Martin et al*.* [6] noted that the role of ethnoveterinary medicine in livestock development is beyond dispute.

In traditional societies around the world, particularly in developing countries, a lot of people have a close relationship with livestock. Livestock is an important and integral part of many economies of these nations [7], which span from the social to the economic to the environmental [8]. In Ethiopia, livestock is a vital and basic component of agricultural production. The country is among the leading countries in Africa in livestock population, with 65 million cattle, 40 million sheep, 51 million goats, 8 million camels and 49 million chickens [9]. It contributes up to 40% of agricultural gross domestic product (GDP), nearly 20% of total GDP and 20% of national foreign exchange earnings [10]. Livestock is a major source of animal proteins, food security, transport of goods, export products, manure for household energy and means of wealth accumulation. It also provides draught power and manure for crop cultivation, which improve soil fertility and aid productivity [11, 12].

However, the livestock sector is constrained by many factors, such as rampant livestock diseases that affect livestock productivity, the marketability of their products and economic losses [13, 14]. Livestock diseases negatively affect the income and farming activities of the rural poor, which in turn has implications for the livelihood of the farmers [15, 16]. It remains one of the principal causes of poor livestock performance, leading to an ever-increasing gap between supply and demand for livestock products [17]. The impact is highly manifested in cultures where livestock is equated with wealth [17]. This is directly related to low accessibility to modern livestock health care, veterinarians and the supply of drugs, even if they are accessible but not affordable to the majority of farmers [18]. As a result, medicinal plants are frequently used to treat different livestock diseases, particularly, in areas where modern veterinary services are absent, expensive, inaccessible and scanty [15, 16, 18].

In Ethiopia, Ethnoveterinary service is believed to have been in practice since time immemorial. The diverse ethnolinguistic communities in Ethiopia are very familiar with the therapeutic potential of medicinal plants as ethnoveterinary medicines. Traditional remedies are major sources of therapeutics for nearly 90% of the livestock population in Ethiopia, and of all forms of traditional medicines in the country, 95% are made of medicinal plants [19]. Thus, traditional healers are potentially important first-line health care providers because they often primarily rely on plant remedies to treat livestock ailments with cheap payment or free of charge in the absence of modern veterinary services [20]. This makes ethnoveterinary practices an integral part of primary health care, especially for marginalized and poor communities living in remote rural areas. Interestingly, practice varies from one place to another and dictated by the diverse culture and tradition of the people as well as the vegetation of a particular area [15]. In Ethiopia, ethnoveterinary medicinal plants are used to treat commonly encountered livestock diseases such as anthrax, blackleg, diarrhoea, wounds, bloat, intestinal worms, external parasites and mastitis [14, 21]. Tilahun et al. [21] stated that the predominant medicinal plants in Ethiopian ethnoveterinary medications are *Croton macrostachyus*, *Solanum incanum*, *Calpurnia aurea* and *Withania somnifera.*

On the other hand, ethnoveterinary knowledge and medicinal plants are at risk of extinction due to ecological and technological changes, access to modern health facilities and anthropogenic and natural factors that threaten the existence of many plant species of veterinary importance [5, 22]. Its mode of transfer and documentation is mainly oral and apprenticeship specific, which leads to the risk of losing such important knowledge that cannot be regained [3, 22]. In addition, rapid socio-economic and outward rural migrations and the paucity of research on the ethnoveterinary uses of medicinal plants in treating livestock diseases further undermine its relevance [23]. Hence, there is consequently a need to scientifically record, document, promote the use of ethnoveterinary medications and the conservation of ethnoveterinary medicinal plants [19]. Such studies can aid in proposing effective and cheaper treatment alternatives to veterinary diseases, enhance ethnoveterinary medicinal plant conservation and provide information for experimentation studies in search of modern pharmaceuticals [2, 5, 24]. These and other related issues have enhanced ethnoveterinary medicine inventories in recent years in Africa [25, 26], and similarly, in Ethiopia [15, 19, 27–33].

The study District is situated in the Central Rift Valley of Ethiopia, between the eastern escarpment and the western escarpment [34, 35], with relatively high biodiversity, but is under great pressure due to agricultural economic corridors and highway constructions, among others. The District has a high livestock population of cattle, sheep, goats and donkeys under the traditional animal husbandry system. In addition to modern veterinary services, local communities use ethnoveterinary medicinal plants and associated traditional knowledge to sustain the health of their livestock. But these knowledge and medicinal plants are becoming fragile due to environmental change, overuse, acculturation, weakening of social structures and verbal modes of knowledge transfer, which lead to information loss and threaten medicinal plants. Consequently, there is a need to document the available indigenous ethnoveterinary knowledge and practices with their respective medicinal plants. In fact, several ethnoveterinary studies have reported veterinary uses of Ethiopian medicinal plants, but compared to the rich cultural and plant diversity in the country, it is still not at the expected level, and to the best of our knowledge, no ethnobotanical research has yet been done on ethnoveterinary practices and medicinal plants in Dugda District. Therefore, the current research aimed (1) to document ethnoveterinary uses of medicinal plants to fill the gap and identify the most potential ethnoveterinary medicinal plants, (2) to quantify consent on ethnoveterinary practices, healing potential and multipurpose ethnoveterinary medicinal plants using relevant ethnobotanical indices and (3) to find new ethnoveterinary medicinal plants used in Dugda District, which could be important source for the discovery of new veterinary pharmaceuticals.

## Materials and methods

### Description of the study area

**Fig. 1:**
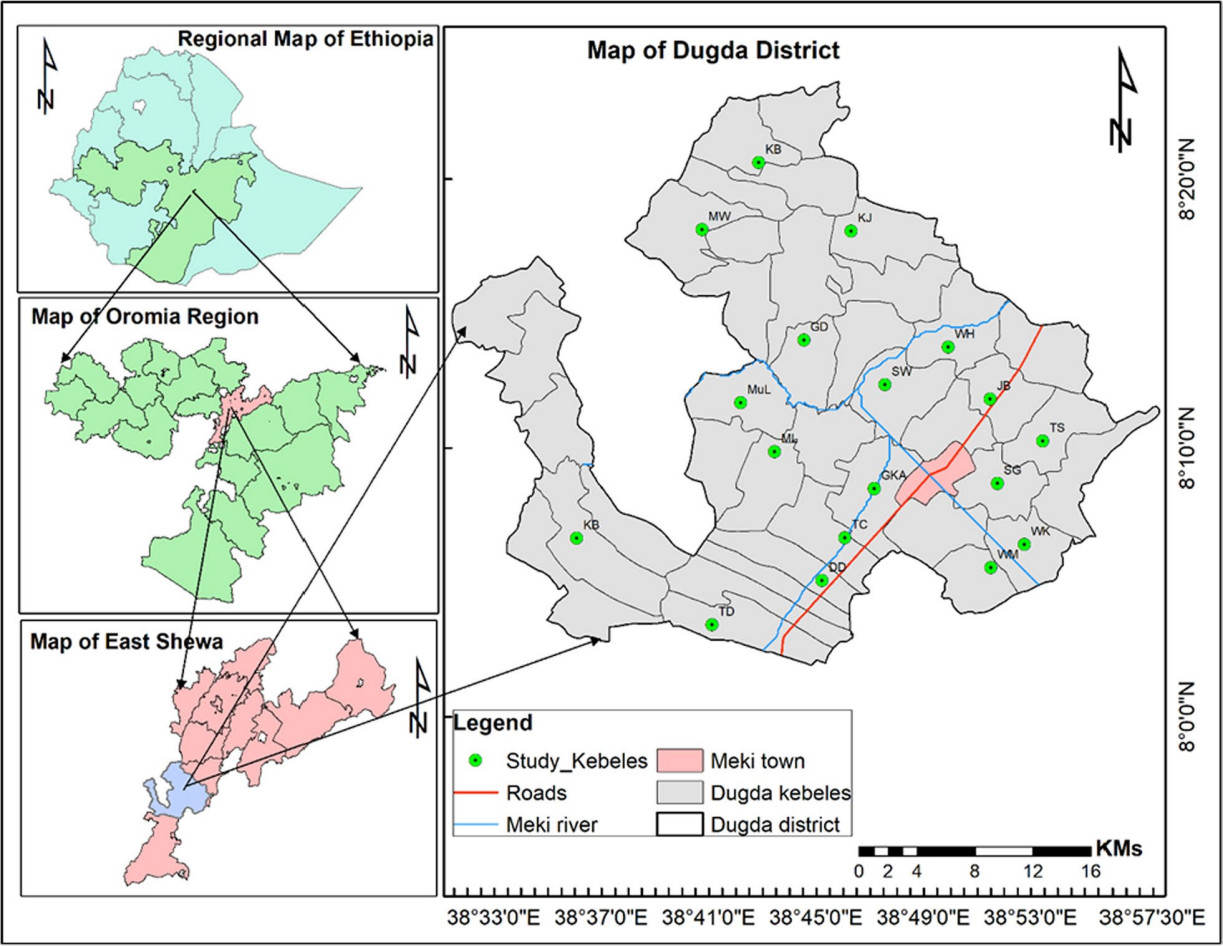
Map of Ethiopia showing Oromia region and the location of the study area (Developed using ArcGIS 10.4.1) Study kebeles are indicated by initials in the map as DD Dodota Danbal, GD Gusa Dongorota, GKA Giraba Korke Adi, JB Jawe Bofo, KiB Kiltu Bilbila, KB Koto Biliti, KJ Koye Jajaba, MJ Maja Lalu, MW Manjikso Waji, MuL Mukiye Laman, SW Sera Wakale, SG Shubi Gamo, TC Tepho Choroke, TD Tuchi Danbal, TS Tuchi Sumayan, WH Walda Hafa, WK Walda Kalina and WM Walda Makdala

The research was conducted in Dugda District located within the Central Rift Valley, Oromia National Regional State, Ethiopia. The study area was selected based on presence of rich indigenous knowledge (*Gada* system), other cultural factors and pressure on remnant woodlands and bushlands patches due to demand for farmlands, irrigation and wood products for different purposes such as charcoal making and collection of fuelwood and construction materials. The study area topographically lies within agro-climate zone called sub-tropical (Weina Dega/Badda-daree) and semi-arid. The landscape is characterized by flat topped plain, valley, mountains and grouped under drought prone highland. The major soil types are sandy loam and clay loam, which have good drainage capacity suitable for irrigation and rain fed crop production. The total area covered by the District is 95,945 ha and situated at 8°01′ N—8°10′ N latitude 38°31′ E—38^°^57′ E longitude and with an altitude 1600–2020 m above sea level (Fig. 1). The average annual rainfall varies from 700 to 800 mm and the temperature ranges from 22° to 28 °C; such rainfall and temperature patterns are relatively, suitable for crop production, animal husbandry and human habitation. Ethnographically, the indigenous people inhabiting the study area belong to the Oromo known as Jille Oromo, the largest ethnic group in Ethiopia, and speak Afaan Oromo, and people with different ethnic backgrounds have also settled there. Mostly the local people are farmers, as a result their economy is based on mixed crop-livestock farming system and fishing. The main agricultural activities are rain fed grain/cereal crops production, irrigation for vegetables and fruit production and livestock farming. The indigenous communities have long tradition of livestock husbandry, particularly, cattle have special place, as key for local economy, source of their livelihood and cultural values. The District is rich in livestock population, with 323,129 cattle, 98,239 sheep, 87,764 goats, 24,194 donkeys, 6,390 horses and 2,645 mules and as well as 251,974chicken (Dugda District Agricultural Office, unpublished data of 2021). The economically important livestock diseases in the District were anthrax, blackleg, bacterial diseases, lumpy skin disease (LSD), Newcastle disease and African horse sickness (AHS) (Dugda District Agricultural Office, unpublished data of 2021). Regarding veterinary services in the District, there are 14 Type D veterinary clinics to service 38 rural kebeles and only one Type B clinic at the District centre Meki town. A total of 32 veterinarians are delivering veterinary services in the District, which include Doctor of Veterinary Medicine (5), Bachelor in Veterinary Science (10), Animal Health Assistances (16) and Veterinary Laboratory Technician (1) (Dugda District Agricultural Office, unpublished data of 2021). The vegetation of the area is described as *Acacia-Commiphora* woodland and bushland land (ACB) under subdivision *Acacia* wooded grassland of the Rift Valley (ACB/RV), mostly dominated by *Acacia* spp. including *Vachellia tortilis, Senegalia senegal, Vachellia seyal* and *Balanites aegyptiaca* [34–36]. This *Acacia* dominated woodland is a highly fragile ecosystem adapted to semi-arid conditions with erratic rainfall, growing on complex and vulnerable hydrological system. The District also has wetland plants such as *Setaria geminata*, *Typha domingensis*, *Cyperus papyrus*, *Nymphaea nouchali* and *Aeschynomene elaphroxylon* [36]. Vegetation of the study area, particularly woodlands and wetlands, are the mainstay of local communities, being vital sources of fuelwood, construction materials, fodder, shade, medicinal plants, wild food plants, farm implements and honey flora. For example, *V. tortilis* for charcoal and firewood; *T. domingensis* for thatching, *A. elaphroxylon* for firewood, roofing and utensils; and *S. senegal* and *Faidgerbia albida* are used for fencing. And some big trees and shrubs like *Ficus sycomorus*, *Solanum incanum*, *Podocarpus falcatus*, *Premna schimperi*, *Ficus vasta* and *V. tortilis* are also socio-culturally important as symbol, sacred and spiritual connections.

### Gada system, livestock and plant conservation

Jille Oromos had traditional governance called *Gada* system, which deals with all-encompassing social, economic, cultural and political activities. Just like other Oromos in different parts of Ethiopia, Jille Oromos have five *Gada* party in their *Gada* system including *Birmaji*, *Melba*, *Mudana*, *Halchisa* and *Robale*, where all have their own leader called *Abba Gada*, transfer power peacefully and each lead for eight years. But before transfer of power each Gada parts have to learn laws and fetch those laws by moving to *Oda Nabe* (near Bishoftu city) with their livestock mainly cattle (*Caffe Godansa*). These laws include law of God (*Sera Waqa*), laws of land (*Sera Lafa*), laws of father and mother (*Sera Abba fi Hadha*) and laws related to other socio-cultural practices. *Abba Gada* has advisors called *Ayyantu* (*Raga*), in which they consult these reputable persons in their *Gada* period for each and every activities. *Ayyantu* is respected person in the society that had God given ability to forecast good fortune and challenges that would happen a year or in eight years of each *Gada* period. These situation could be related to an individual or the whole society, such as rainy seasons of the year, livestock diseases, war/conflict, peace, drought and famine. In doing this, they have established a kind of trend that would happen in each *Gada* period, for example, in *Gada Birmaji* there would be high frequency of livestock diseases, *Gada Melba* known by conflict and truth, *Gada Mudana* by plenty of rain and prosperity, *Gada Halchisa* good for livestock productivity and *Gada Robale* known by drought and famine. Thus, Gada system had important role in livestock production and welfare, based on each circumstances in each *Gada* period these would make livestock keepers be prepared beforehand, to locate alternative grazing lands and water resources for their livestock.

Furthermore, *Gada* system had a concept of plant conservation, by prohibiting cutting of respected plants and protection of places of *Gada* practices (Arda Jila/Malka). These plant species include *Ficus sycomorus* (Oda), *F. thonningii*, *Podocarpus falcatus*, *Premna schimperi*, *Phoenix reclinata* and *Cordia Africana*. The aforementioned plant species and others are also important in *Gada* practices as sources of material cultures, constructions, ritual grounds, praying and blessings. One typical example of sacred place in the study area is a place called *Oda Tuta* (group of *F. sycomorus* trees), where over 200 *F. sycomorus* stands found in one place and serve for various *Gada* events. However, nowadays such practices are weakening due to various factors and need attention and revitalization for plant biodiversity and associated indigenous knowledge conservations.

### Participant selection and interviewing process

Ethnoveterinary survey was conducted during several field trips made between April 2020 and June 2021 in 18 kebeles of Dugda District. The reconnaissance survey was carried out prior to data collection and the researchers got overview on vegetation types, ethnoveterinary knowledge of medicinal plants, knowledgeable people, natural resource management and conservation practices. The study kebeles were selected with the help of Development Agents, local leaders, elders and observation during the reconnaissance survey, based on the vegetation cover and availability of knowledgeable people on ethnoveterinary medicinal plants (Fig. 1). The key informants were selected through purposive and snowball techniques [37]. The representative general informants were selected through systematic random sampling techniques following the methods described by Martin [38]. During selection of informants different socio-demographic characteristics of the informants were considered. A total of 378 informants (from 18 kebeles (subdistricts or smallest administrative units in Ethiopian Admin system), 21 informants each) were sampled, where general informants (288) were selected in volunteer and interviewed, during field trips made in the respective study kebeles. Nominations on knowledgeable persons on medicinal plants to participate as key informants was made by the help of elderly people, Development Agents and village leaders in the study kebeles. Following this, a total of 90 traditional healers (27 female and 63 male) were selected. These healers have high reputation with respect to their traditional knowledge on medicinal plants and long-term ethnoveterinary services. Regarding gender of informants’, male informants were 250, while 128 informants were female. The age of the informants’ ranges between 20 and 87 years. They are distributed into two age groups, 131 informants (20–39) young group and 247 informants (40–87) matured group, where 40 years old is considered as turning into maturity in knowledge and leadership. The majority of the interviewed informants were illiterate (257, 67.99%), who did not attend school. And occupationally, most informants were farmers (313, 82.80%), who’s daily livelihood mainly based on agricultural activities like crop production and livestock farming (Table 1).

**Table 1:** Demographic characteristics of the informants

Parameter used	Category of informants	Number of informants	Percentage
Gender	Male	250	66.14
	Female	128	33.86
Age	Young group (20–39 years)	131	34.66
	Matured group (40–87 years)	247	65.34
Informant category	General informant	288	76.19
	Key informant	90	23.81
Educational level	Illiterate	257	67.99
	Literate	121	32.01
Occupation	Farmers	313	82.80
	Others (students, craft, merchant and fishing)	65	17.20

### Ethnobotanical data collection

Ethnoveterinary medicine data were collected using semi-structured interviews, focus group discussions, participant field observations and with very close interaction with informants. The semi-structured interview in the field survey with informants were conducted in local language called “Afaan Oromo” following method described in Martin [38]. The appropriate ethical permission to conduct the research was obtained through formal letter by Addis Ababa University (AAU) to Dugda District authority and local community leaders (Kebeles). Before interview process, government bodies at district and kebele levels, and all informants were briefed about the research objectives and its academic purposes. Indeed, prior informed consent was obtained verbally from each informant before the commencement of interviews, then semi-structured interviews and focus group discussions were conducted. The semi-structured interview contained questions on socio-economic and demographic characteristics regarding name, age, sex, level of education, occupation, religion and ethnicity of informants. Information regarding local names of medicinal plants, ailments treated, habitats of the species, degree of management (wild/cultivated), part/parts used, conditions of plant part used (fresh/dried), other ingredients or additives, methods of remedy preparations, routes of remedy administration, noticeable adverse effects of remedies, use of antidotes for adverse effects, taboos/beliefs related to collection and use of plant, source of knowledge, methods of indigenous knowledge transfer, other use of medicinal plants, existing threats and traditional conservation practices (if any) were collected following ethnobotanical methods described in [16, 19, 39]. Further, focus group discussions (6–8 informants) in each study kebeles (subdistrict) were conducted to validate, clarify and harmonize the results obtained through semi-structured interviews [40]. Besides, guided field observations were performed with key informants which create an opportunity for more discussion on different issues, and useful in describing and practical identification of medicinal plants, identifying different vegetation types and land-use impacts by indicating problems or possible solutions following the method described by Cunningham [41]. Finally, informants’ description of livestock ailment types was translated with the consultation of veterinarians working in the study area and then translated into veterinary terms.

Voucher specimens of the medicinal plant cited for their ethnoveterinary services were collected and identified at least in their local names with the help of key informants during guided field observations, following standard botanical collection procedures. Plant voucher specimens were collected from natural vegetation, woodlands, grasslands, farmlands and home-gardens during the field walks with the help of informants and local field assistants. Voucher specimen identification was done in the field and later at the National Herbarium (ETH) of Addis Ababa University (AAU) using taxonomic keys provided in the relevant volumes of Flora of Ethiopia and Eritrea [42–48]. The specimen identification was confirmed by a plant taxonomic expert at AAU. The identified voucher specimens were numbered, labelled and deposited in the ETH. Plant scientific names were updated according to the World Flora Online (WFO, http://www.worldfloraonline.org) and databases such as PubMed, Google Scholar, ScienceDirect and SpringerLink were used for literature collection and compiled in Zotero, then compared with the present study.

### Quantitative ethnobotanical data analysis

Ethnoveterinary data were arranged and organized by using Microsoft Excel spreadsheet software (Microsoft Excel, 2013). Quantitatively analysed using descriptive statistic methods like percentile and frequency distribution were used to summarize medicinal plant data based on use reports. The raw data of ethnoveterinary medicinal species were summarized and reported in table form with their scientific name, family name, local name, growth form, ailment treated, plant parts used, ailments, livestock types treated, conditions of plant part used, method of preparation and application, route of administration and collection number. The statistical test of significance was performed using independent sample *t* test on the number of medicinal plants cited by different groups: gender (male and female), age (young and matured), informant type (key and general informants) and educational level (illiterate and literate). The analysis of quantitative ethnobotanical data was conducted using informant consensus factor (ICF), fidelity-level (FL) and relative importance (RI) formulas as described below.

### Informant consensus factor (ICF)

The informant consensus factor (ICF) values was used to assess the homogeneity or degree of agreement of the informants’ knowledge about medicinal plants, which help to determine the most important livestock ailment categories in the study area. It is calculated as follows:

$${\text{ICF}}\, = \,\left( {{\text{Nur}}\, - \,{\text{Nt}}} \right)/{\text{Nur}}\, - \,{1})$$

where n = number, and UR = use report (s), while t = plant (s) [39, 49, 50].

### Fidelity level (FL)

Fidelity level (FL) of the medicinal plants conducted to compare and determine relative healing potential of medicinal plants based on ailment category. It was computed as follows:

$${\text{FL}}\left( \% \right)\, = \,{\text{Ip}}/{\text{Iu}}\, \times \,{1}00$$

where Ip is the number of informants who independently cited ailment category and Iu the total number of informants who reported the plants for any given ailment category [51].

### Relative importance (RI)

Relative importance (RI) of each cited medicinal plant was calculated using a method by Bennett and Prance [52]. RI was calculated using:

$${\text{RI}}\, = \,{\text{NP}}\, + \,{\text{NCS}}$$

where NP is obtained as: number of specific ailments treated by a given species divided by the total number of specific ailments treated by the most multipurpose species. NCS is the number of ailment categories treated by a given species divided by the total number of ailment categories treated by the most multipurpose species. Species with RI value close to 2 are the ones with the highest diversity of medicinal application and close to 0 least diversity of medicinal application.

## Results

### Major livestock diseases in the study area

A total of 37 livestock ailments have been reported by informants, which are treated by 64 ethnoveterinary medicinal plants. The ailments of livestock in the District were mostly seasonal, which made the livestock susceptible during seasonal transition as result of changes in vegetation and animal fodders composition. The most prevalent and commonly treated ailments were anthrax with 18 species (28.13%), followed by inappetence (15 species, 23.44%), diarrhoea (13, 20.31%), rabies (12, 18.75%), bloating (11, 17.19%), blackleg and mange (10, 15.63%) each. Other important animal ailments were treated by 3 to 7 medicinal plants as indicated in Table 2.

**Table 2:** Ailments treated by ethnoveterinary medicinal plants

Livestock ailments	Livestock type affected	No. of species	% of species
Anthrax	Cattle	18	28.13
Inappetence	Cattle	15	23.44
Diarrhoea	Cattle	13	20.31
Rabies	All livestock	12	18.75
Bloat	Cattle	11	17.19
Blackleg	Cattle	10	15.63
Mange/Alopecia	Cattle	10	15.63
Bovine ephemeral fever (BEF)	Cattle	7	10.94
Colic	Cattle	5	7.81
Prophylaxis against rabies	Dogs and cats	5	7.81
Eye disease	Cattle, goats and sheep	4	6.25
Wound	Cattle, goats and sheep	4	6.25
Retained placenta	Cattle, goats and sheep	4	6.25
Fattening and strengthening	Cattle	3	4.69
When a cow refuses its calf	Cattle	3	4.69

### Taxonomic diversity ethnoveterinary medicinal plants

**Fig. 2:**
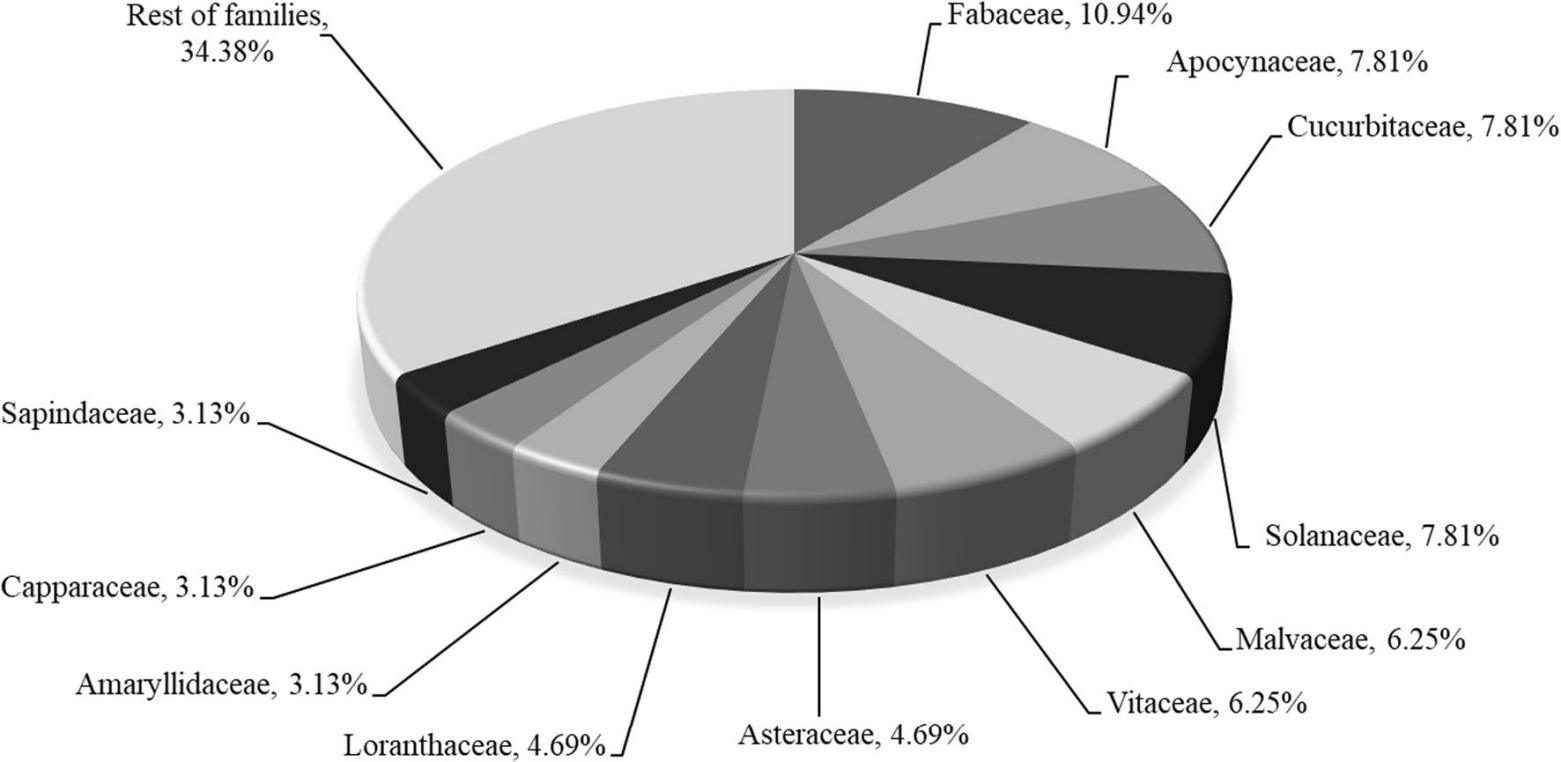
Distribution Ethnoveterinary medicinal plants of Dugda District in botanical families

In this study, a total of 64 ethnoveterinary plants belonging to 58 genera and 33 families were reported to treat different livestock ailments. Of the total, the majority of plant species (52) were native or indigenous to Ethiopia and a single plant species was recorded as endemic (*Kalanchoe petitiana*). In addition, among total ethnoveterinary medicinal plants, 11 (17.19%) plant species were introduced and five plant species (7.81%) were identified as invasive alien species (Tables 9 and 10). The analysis of botanical families of ethnoveterinary medicinal plants showed that 33 plant families were involved in ethnoveterinary practices of Dugda District. Of the total plant families, 65.62% of medicinal plants were contributed by eleven plant families, where as 34.38% come from the remaining families. Family Fabaceae was the best represented, with seven medicinal plant species, followed by Apocynaceae, Cucurbitaceae and Solanaceae, with five species each, Malvaceae and Vitaceae, with four species each (Fig. 2). The results from life forms of ethnoveterinary medicinal plants indicated that herbs were the most frequently utilized life forms in ethnoveterinary services of the study area, with 21 species (32.81%), and followed by shrubs 20 (31.25%). The other therapeutic life forms utilized in remedy preparation were climbers 13 (20.13%) and trees 10 (15.63%).

### Plant parts used for remedy preparation

**Fig. 3:**
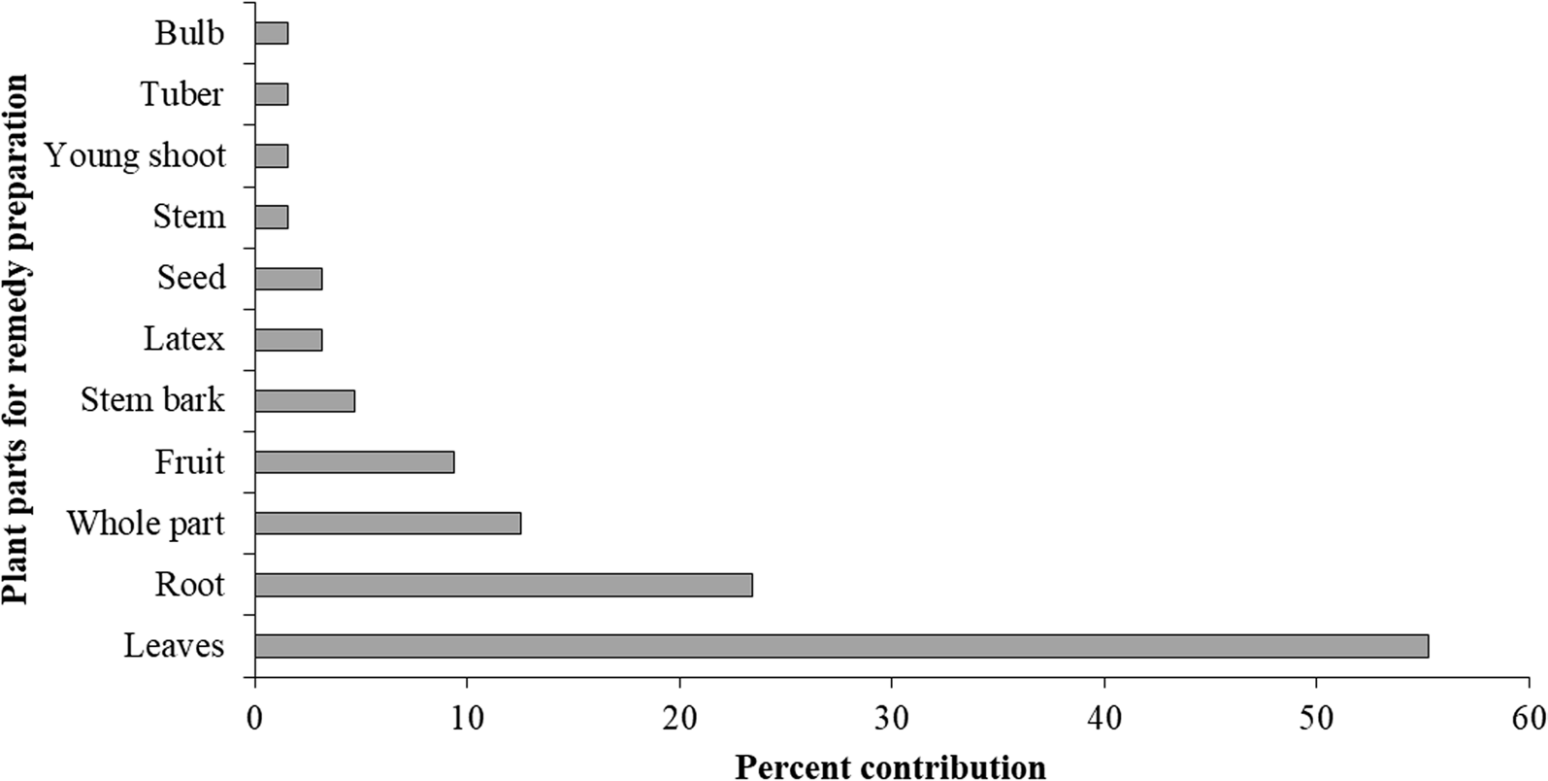
Plant parts used for remedy preparation in Dugda District

The analysis of plant parts used for ethnoveterinary remedy preparations revealed that leaves were the most frequently used plant parts, which come from 36 species (56.25%), followed by root (15 species, 23.44%), whole part (8, 12.50%) and fruits (6, 9.38%). In addition, other plant parts such as stem bark, latex, seeds, bulbs, stem, tuber and young shoot were also sought for remedy preparations in ethnoveterinary practices of the study District, with low percent of contribution (Fig. 3). Regarding condition of plant parts during remedy preparations, the majority (92.19%) of remedies were prepared from freshly harvested plant parts and a few preparations from fresh/dry (10.94%) and dry (9.38%) plant parts.

### Modes of remedy preparation and application

**Fig. 4:**
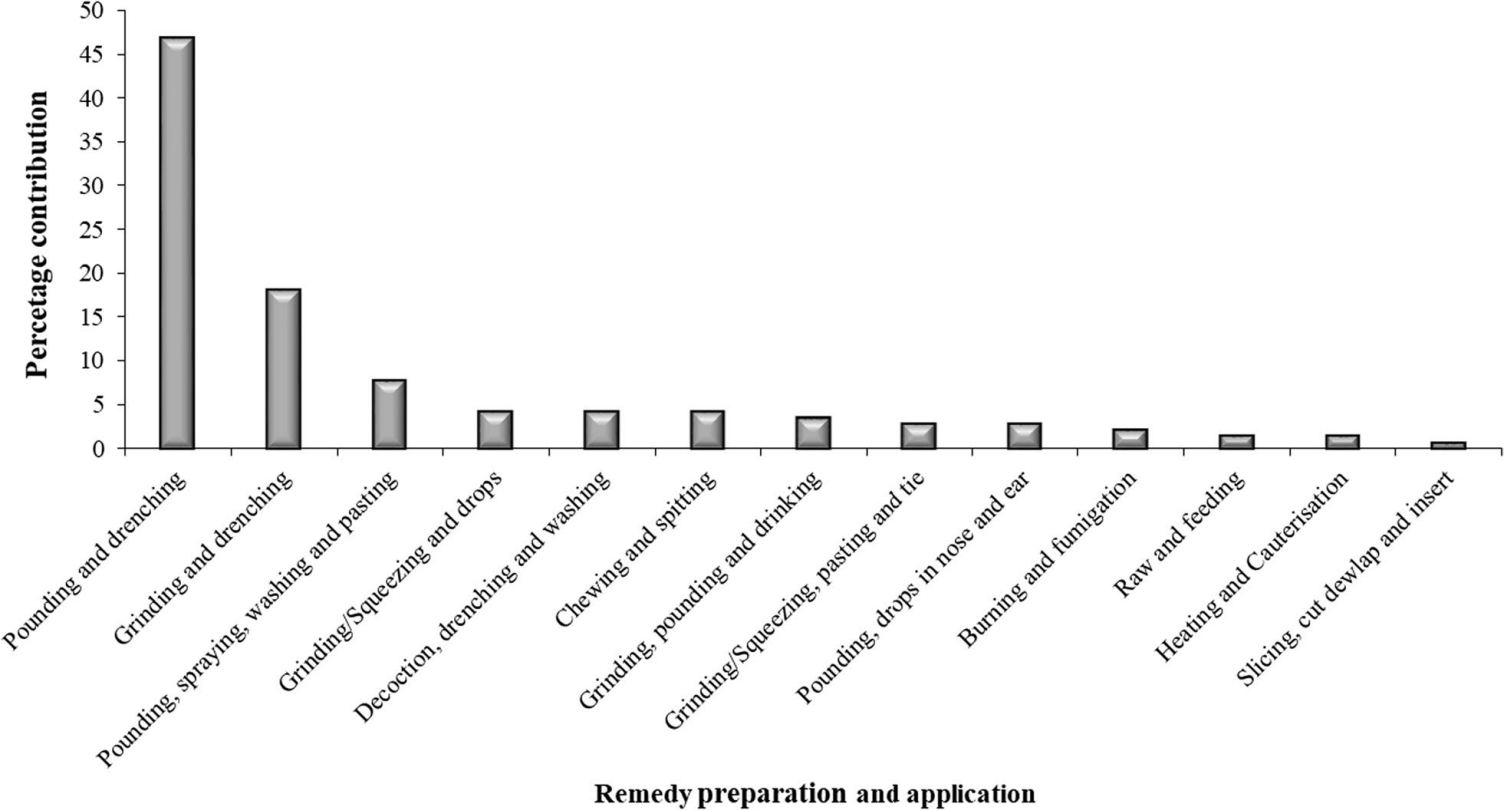
Modes of ethnoveterinary remedy preparation and application in Dugda District

The local people and traditional healers of the Dugda District have reported several traditional methods used in preparation and application of ethnoveterinary remedies for treatment of various ailments in livestock. Among these modes of preparation and application, pounding and drenching (46.85%) was the most common mode of remedy preparation and application, followed by grinding and drenching (18.18%), and pounding, spraying, washing and pasting (7.69%) (Fig. 4). Pounding, best way of preparation in the area, where plant parts as mono-preparation or poly-herbal preparation are pounded using a wooden mortar and pestle, then soaked in cold water to get intended veterinary remedies. The majority of ethnoveterinary remedies (84.31%) were formulated from single plant species, but only 15.69% of the remedies were made from poly-herbal (multi-plants) formulations, i.e. ethnoveterinary remedies prepared from concoction of two or more medicinal plant species (Tables 9 and 10). For instance, poly-herbal preparation from concoction of eight medicinal plants; roots from *Cyphostemma cyphopetalum*, *Withania somnifera*, *Cucumis ficifolius, Foeniculum vulgare*, and leaves from *Dodonaea viscosa* subsp. *angustifolia*, *Gymnanthemum amygdalinum* (Synonym: *Vernonia amygdalina*), *Marsdenia schimperi*, (Synonym: *Dregea schimperi*), and young shoots of *Croton macrostachyus*. These all medicinal plant parts were reported to be pounded together, mixed with cold water and salt, being effective remedy for cattle ailments, namely anthrax, blackleg, LSD and bovine ephemeral fever (BEF). In either ways, cold water was the most frequently used solvent to extract bioactive ingredients found in ethnoveterinary medicinal plants of the study area. After preparation, the traditional healers administer remedies to diseased livestock as drenches without sieving. In addition to plant materials and water, some other ingredients were also used in formulations of remedies, these include: salt, soot/charcoal, used petrol, kerosene, food, saliva, milk, blood, butter/ghee, whey and skimmed milk.

On top of all, the important process in ethnoveterinary medicine or ethnomedicine practice is locating sources of medicinal plants by practitioners. In the study area, local people and traditional healers travel in search of fresh ethnoveterinary medicinal plants in different habitats/places including woodlands, parklands, farmlands, farm margins, fence, homestead, wetlands, riverine, on host trees for mistletoes and bought from markets, as the need of ethnoveterinary medicines arise. The data on sources of medicinal plants showed that indigenous the people of the study area mostly utilize wild medicinal plant resources (53 species), while a few (11 species) medicinal plants were recorded as cultivated and semi-wild, six and five medicinal plant species, respectively.

### Routes of ethnoveterinary remedy administration

**Fig. 5:**
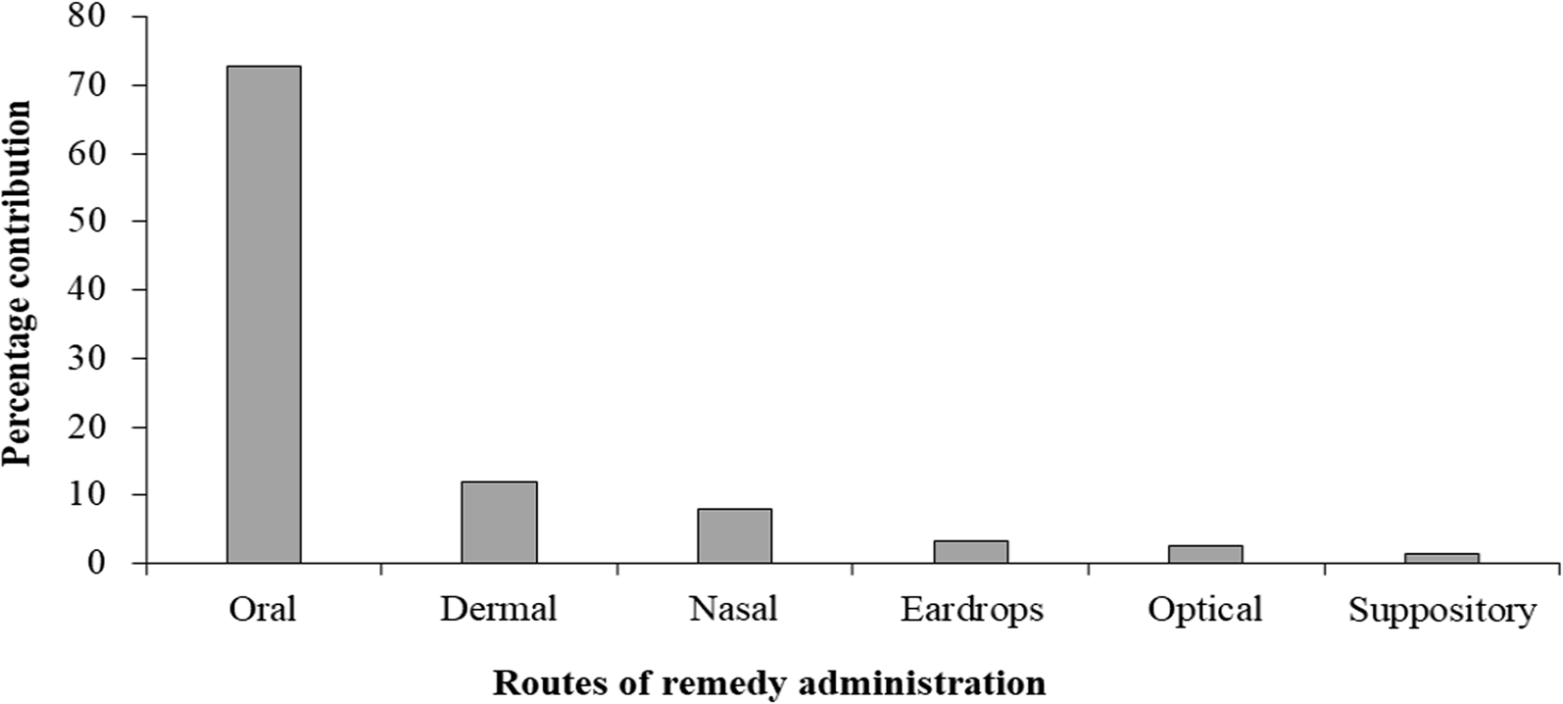
Routes of administration of ethnoveterinary remedy in Dugda District

Ethnoveterinary remedies for livestock in Dugda District were administered through different administration routes based on encountered ailments. The dominant administration route was oral (72.67%), followed by dermal (12%) and nasal (7%). The remaining routes of administration were less frequently used applications (Fig. 5). Oral application, the most frequently applied route of remedy administration, is practiced as drenching herbal remedy through mouth of the diseased livestock.

In connection with this, farmers and traditional healers determine the dosage of remedies, after visual inspections of the diseased animals, on the basis of age, sex, physical condition of the diseased animals, severity of the disease, types of animals and pregnancy status of animals. The veterinary remedies were measured by variety of instruments such as finger length, Can, glass, plastic jug, coffee cup, bottle, number of drops and number of plant heads. Although there is no standardized dose, a wide ranges of doses were applied for treatment of livestock diseases, starting from single drop via left nostril and ear of dogs and cats (prophylaxis against rabies) to two Can (approximately two litre) to treat diseases like anthrax, blackleg, BEF and LSD. Similarly, the administration frequency of remedies delivered to the diseased animals ranges from only once to until the animals recovered from the disease.

### Ethnoveterinary uses of medicinal plants and types of livestock treated

Ethnoveterinary uses of medicinal plants in the study area were analysed based on their use reports. The most utilized medicinal plant species include: *W. somnifera* (53 use reports), *Kedrostis foetidissima* (43), *C. cyphopetalum* (35), *Gymnanthemum amygdalinum* (35), *Cucumis ficifolium* (34), *Calpurnia aurea* (33), *Senna occidentalis* (32), *Phytolacca dodecandra* (28), *Croton macrostachyus* (28), *Melia azedarach* (25), *Cyphostemma pannosum* (22), *Dodonaea viscosa* subsp*. angustifolia* (22) and *Foeniculum vulgare* (20) (Table 3).

**Table 3:** Ethnoveterinary medicinal plants with the highest use reports

Ethnoveterinary medicinal plants	Livestock ailments treated	Urs
*Withania somnifera*	Gastrointestinal disorders (Blo, Col, Dia, Ant), Dermatological disorders (Man, LSD), Musculoskeletal disorders (Bla) and General illness (Eve)	53
*Kedrostis foetidissima*	Gastrointestinal disorders (Blo, Dia), Dermatological disorders (Lym), External parasites (Lic), Reproductive disorders (Rep) and general illness (Fat & Str, BEF)	43
*Cyphostemma cyphopetalum*	Gastrointestinal disorders (Blo, Col, Ant), Dermatological disorders (LSD), Musculoskeletal disorders (Bla), General illness (BEF) and Neurological disorders (Rab)	35
*Gymnanthemum amygdalinum*	Gastrointestinal disorders (Dia, Blo, Col, Ant), Musculoskeletal disorders (Bla), Dermatological disorders (LSD) and General illness (Evs, BEF)	35
*Cucumis ficifolius*	Gastrointestinal disorders (Blo, Dia, Ant), Musculoskeletal disorders (Bla), Dermatological disorders (LSD, Man) and General illness (BEF)	34
*Calpurnia aurea*	External parasites (Li, Tic) and Neurological disorders (Rab) and Gastrointestinal disorders (Ant)	33
*Senna occidentalis*	Dermatological disorders (Snbi, Snbr), Gastrointestinal disorders (Col, Dia, Ant, Blo) and General illness (BEF)	32
*Croton macrostachyus*	Musculoskeletal disorders (Bla), Dermatological disorders (LSD, Man), Gastrointestinal disorders (Dia, Ant, Blo), Neurological disorders (Dul) and General illness (BEF)	28
*Phytolacca dodecandra*	Dermatological disorders (Riw, Act, FMD), Gastrointestinal disorders (Con), General illness (BEF), Ophthalmologic disorders (Eyd) and Neurological disorders (Rab)	28
*Melia azedarach*	Gastrointestinal disorders (Col, Blo, Dia) and General illness (Inp, Chd)	25
*Dodonaea viscosa* subsp. *angustifolia*	Musculoskeletal disorders (Bla), Dermatological disorders (LSD, Man), Gastrointestinal disorders (Ant) and General illness (BEF)	22
*Cyphostemma pannosum*	Dermatological disorders (Man) and General illness (BEF, eqd)	22
*Foeniculum vulgare*	Dermatological disorders (LSD, Man), Musculoskeletal disorders (Bla), Gastrointestinal disorders (Ant), Reproductive disorders (Urr) and General illness (BEF)	20
*Achyranthes aspera*	Gastrointestinal disorders (Dia), Musculoskeletal disorders (Bla), Dermatological disorders (Man) and General illness (Eqd)	19
*Marsdenia schimperi*	Musculoskeletal disorders (Bla), Dermatological disorders (LSD, Man), Gastrointestinal disorders (Ant) and General illness (BEF)	16
*Calotropis procera*	Musculoskeletal disorders (Bla) and Dermatological disorders (War, Wou)	15
*Cissus quadrangularis*	Gastrointestinal disorders (Ant)	15
*Verbascum sinaiticum*	Gastrointestinal disorders (Dia, Blo, Col), Dermatological disorders (Man, Sca), Respiratory disorders (Pas), Neurological disorders (Rab) and General illness (Inp)	15
*Oreosyce africana*	Dermatological disorders (Man), Gastrointestinal disorders (Ant) and General illness (AHS)	14
*Solanum incanum*	Respiratory disorders (Cou, Nad) Dermatological disorders (Riw, Mod)	13

Gastrointestinal disorders (Blo = Bloat, Col = Colic, Dia = Diarrhoea, Ant = Anthrax, Con = Constipation), Dermatological disorders (Man = Mange, LSD = Lumpy skin disease, Lym = Lymphangitis, Snbi = Snake bite, Snbr = Snake breathe, Riw = Ringworm, Act = Actinomycosis, FMD = Mouth and foot disease, Sca = Scabies, War = Warts, Wou = Wounds, Mod = Mouth disease), Musculoskeletal disorders (Bla = Blackleg, Bof = Bone fracture), General illness (Eve = Evil eye, Fat & Str = Fattening and Strengthening, BEF = Bovine ephemeral ever, Evs = Evil spirit, Inp = Inappetence, Dul = Dullness, Chd = Chicken disease, Eqd = Equine disease, AHS = African horse sickness), External parasites (Lic = Lice, Tic = Ticks), reproductive disorders (Rep = Retained placenta, Urr = Urine retention), Neurological disorders (Rab = Rabies), Ophthalmologic disorders (Eyd = Eye disease), Respiratory disorders (Cou = Cough, Nad = Nasal discharge, Pas = Pasteurellosis)

Ethnoveterinary medicines, prepared from aforementioned and other therapeutic plant species, are reported to be utilized for the treatment of various ailments of domestic animals. The majority of ethnoveterinary medicinal plants (56 species, 87.50%) were used to treat cattle, followed by goats and sheep (16, 25%), all livestock (12, 18.75%), equines (10, 15.63%), dogs and cats (5, 7.81%), but only single preparation (1, 1.56%) was reported for treatment of chicken disease.

### Ethnoveterinary ailment category

A total of 37 ailments of the livestock were recorded in Dugda District. These ailments were further categorized into nine ailment categories, and then, informants’ consensus factor (ICF) values were calculated for each categories. The output of ICF values ranges from 0.69 to 0.94. The ailment category with the highest ICF value was respiratory diseases (0.94). The other ailment categories were external parasitic diseases (0.91), gastrointestinal diseases (0.88), dermatological diseases (0.85), neurological diseases (0.83) and musculoskeletal diseases (0.81) (Table 4). Even though gastrointestinal diseases ranked at third place next to external parasitic diseases, the ailment category was reported to be treated by high number of medicinal plants with informants citations (34 species, 267 citation), followed by dermatological diseases (26, 168), and general illness diseases (24, 89).

**Table 4:** Informant consensus factor of ethnoveterinary ailment category

Ailment category	Nt	% of all species	Nur	% of all Nur	ICF
Gastrointestinal diseases	34	53.13	267	36	0.88
Neurological diseases	12	18.75	66	8.98	0.83
Musculoskeletal diseases	14	21.88	71	9.66	0.81
External parasitic diseases	3	4.69	23	3.13	0.91
Dermatological diseases	26	40.63	168	22.86	0.85
Reproductive diseases	6	9.38	17	2.31	0.69
Respiratory diseases	2	3.13	19	2.59	0.94
Ophthalmologic diseases	4	6.25	15	2.04	0.79
General illness	24	37.50	89	2.04	0.74

Nt: Number of plants (s) mentioned for the treatment of these particular disease groups, Nur: Number of times a particular category of diseases is cited, ICF: informant consensus factor

### Medicinal plants used to treat ethnoveterinary ailment category

Relative healing potential of main medicinal plants used to treat each ailment category was evaluated by calculating their fidelity level (FL). Accordingly, the FL values of the three most cited medicinal plants for the treatment of ethnoveterinary ailment category were presented in (Table 5). The results of FL values of medicinal plants showed that there was high degree of agreement (> 50% FL) on selection of medicinal plants used to treat gastrointestinal, dermatological, reproductive, respiratory and ophthalmologic diseases categories, while external parasitic and general illness categories showed low agreement (< 50% FL). For example, high fidelity-level values were obtained for *Melia azedarach* (92%), *Kedrostis foetidissima* (67.4%) and *Croton macrostachyus* (58.8%) to treat gastrointestinal disease category.

**Table 5:** Fidelity-level value of top three medicinal plants commonly reported against ethnoveterinary ailment category

Ethnoveterinary ailment category	Ethnoveterinary medicinal plants	Ip	Iu	FL Value %
Gastrointestinal diseases	*Kedrostis foetidissima*	29	43	67.4
	*Melia azedarach*	23	25	92.0
	*Croton macrostachyus*	20	34	58.8
Neurological diseases	*Phytolacca dodecandra*	16	28	57.1
	*Cyphostemma cyphopetalum*	13	35	37.1
	*Datura stramonium*	8	10	80.0
Musculoskeletal diseases	*Calotropis procera*	15	15	100
	*Balanites aegyptiaca*	8	11	72.7
	*Withania somnifera*	7	53	13.2
External parasitic diseases	*Calpurnia aurea*	18	33	54.5
	*Withania somnifera*	3	53	5.7
	*Kedrostis foetidissima*	3	43	7.0
Dermatological diseases	*Cyphostemma pannosum*	19	22	86.4
	*Achyranthes aspera*	12	19	63.2
	*Kalanchoe petitiana*	11	11	100
Reproductive diseases	*Grewia ferruginea*	5	5	100
	*Pappea capensis*	5	5	100
	*Searsia natalensis*	3	5	60.0
Respiratory diseases	*Solanum incanum*	11	13	84.6
	*Nicotiana tabacum*	8	8	100
Ophthalmologic diseases	*Capparis fascicularis*	5	5	100
	*Verbena officinalis*	4	4	100
	*Gossypium hirsutum*	3	3	100
General illness	*Withania somnifera*	13	53	24.5
	*Gymnanthemum amygdalinum*	7	35	20.0
	*Cucumis ficifolius*	6	34	17.6

Ip: number of informants who independently cited ailment category; Iu: total number of informants who reported the plants for any given ailment category; FL: fidelity level

### Relative importance of ethnoveterinary plants

The relative importance of ethnoveterinary medicinal plants was analysed to identify the most multipurpose (use diversity) medicinal plants based on the number of specific ailments and ailment categories treated by these plants. Accordingly, 27 (42.19%) medicinal plants were used to treat two or more specific ailment; likewise, about 25 (39.06%) medicinal plants were utilized to manage two and more ailment categories. The relative importance analysis (RI) revealed that *Croton macrostachyus* had highest diversity of uses (2), followed by *Kedrostis foetidissima*, *Withania somnifera*, *Phytolacca dodecandra* and *Senna occidentalis* (Table 5). Interestingly, the majority of reported medicinal plants were used as gastrointestinal agents (53.13%), followed by dermatological agents (43.10%), general illness agents (40.63%), musculoskeletal agents (21.88%) and neurological agents (18.75%) (Table 6).

**Table 6:** Relative importance of ethnoveterinary medicinal plants in Dugda District

Plant species	NSA	NAC	NP	NCS	RI value	%
*Croton macrostachyus*	8	5	1	1	2	100
*Kedrostis foetidissima*	7	5	0.875	1	1.875	93.75
*Withania somnifera*	8	4	1	0.8	1.8	90
*Phytolacca dodecandra*	6	5	0.75	1	1.75	87.5
*Senna occidentalis*	7	4	0.875	0.8	1.675	83.6
*Foeniculum vulgare*	5	5	0.625	1	1.625	81.25
*Gymnanthemum amygdalinum*	6	4	0.75	0.8	1.55	77.5
*Cucumis ficifolius*	6	4	0.75	0.8	1.55	77.5
*Cyphostemma cyphopetalum*	6	3	0.75	0.6	1.35	67.5
*Marsdenia schimperi*	4	4	0.5	0.8	1.3	65
*Dodonaea viscosa* subsp. *angustifolia*	4	4	0.5	0.8	1.3	65
*Calpurnia aurea*	5	3	0.625	0.6	1.225	61.25
*Melia azedarach*	5	3	0.625	0.6	1.225	61.25
*Aloe trichosantha*	4	3	0.5	0.6	1.1	55
*Verbascum sinaiticum*	5	2	0.625	0.4	1.025	51.25

NSA = number of specific ailments; NAC = number of ailment categories; NP = NSA treated by a given species divided by the total NSA treated by the most multipurpose species. NCS = NAC treated by a given species divided by the total NAC treated by the most multipurpose species, RI = Relative importance value

### Distribution of ethnoveterinary medicinal plant knowledge

The statistical test of significance was performed using independent sample *t* test on the number of medicinal plants cited by informant groups in the study District. The result of independent sample *t* test revealed that there was significance difference (*P* < 0.05) between key and general informants on the number of medicinal plant species. Similarly, there was significance difference (*P* < 0.05) between the two age groups; young group (20–39 years) and matured group (40–87 years) informants. Significance difference (*P* < 0.05) was also obtained between illiterate and literate informants on the number of medicinal plant species mentioned. However, there was no significance difference (*P* > 0.05) in the number of medicinal plants reported by male and female informants (Table 7).

**Table 7:** Statistical test of significance among different informant groups based on number of reported medicinal plants by informants in Dugda District

Parameters used	Informant groups	N	NMP reported	Mean	SD	*t *value**	*P* value
Informant category	General informant	288	593	2.059	1.767	− 9.962	0.000*
	Key informant	90	413	4.589	2.198		
Gender	Male	250	678	2.712	2.061	0.608	0.544
	Female	128	328	2.563	2.357		
Age	Young group (20–39 yrs)	131	210	1.603	1.429	− 8.47	0.000*
	Matured group (40–87 yrs)	247	796	3.227	2.276		
Educational level	Illiterate	257	775	3.016	2.262	5.258	0.000*
	Literate	121	213	1.909	1.718		

*Significant difference (*p* < 0.05); ** t(0.05) (two tailed), df = 376, N = Number of respondents, NMP = Number of medicinal plants, SD = Standard Deviation, yrs = years

## Discussion

### Livestock ailments and diversity of ethnoveterinary medicinal plants

In Dugda District, similar to other part of Ethiopia, livestock is an important and integral wealth of indigenous people, where livestock provide cash income and foods, means of transportation, draught power, wealth accumulation (*Kila lixu*), socio-cultural values as gift upon marriage (*Gabra/Biti* and *Tirma/Gega’o*), in religious practices (*Fala*), *Gada* system (*Butta qalu*) and other cultural events. However, livestock populations, particularly cattle are frequently affected by livestock ailments including anthrax, inappetence, diarrhoea, rabies, blackleg and mange, which deteriorate health and productivity. These ailments are also among the top prevalent livestock ailment in the study District, which are also among the commonly encountered ailments throughout the country and commonly reported to be treated by herbal medications [14, 21]. Similar ethnoveterinary inventories conducted elsewhere in Ethiopia also showed the prevalence of anthrax, inappetence, diarrhoea, rabies, blackleg and mange, including researches by Yineger et al. [16] at Bale Mountains National Park, Giday and Teklehaymanot [18] in Afar people of Ada’ar District, Kidane et al. [15] in Maale and Ari ethnic communities, Lulekal et al. [19] for people of Ankober District, and Assefa and Bahiru [29] at Abergelle, Sekota and Lalibela districts. Although the outbreak and prevalence of livestock diseases varies among the areas due to climatic differences, the comparison showed similar disease-causing factors, wide prevalence and serious economic impact of these diseases in the country, beside a nearly similar livestock’s management system throughout the country.

Most importantly, a good number of ethnoveterinary medicinal plants (64) are reported to be used in management of livestock ailments in Dugda District, pertinent to availability, cultural acceptability, affordability and efficacy to treat livestock [2]. This is supported by the argument of Van der Merwe et al. [2] who stated that the indications for ethnoveterinary medicines in livestock are similar in different areas, but the ethnoveterinary medicinal plants used are usually different, based on locally available plant populations. In general, indigenous and local people of a given area developed an indigenous and local knowledge system that best fits to select and use diverse curative medicinal plants to treat frequently occurring livestock diseases [19] and less severe diseases and or injuries [53].

Interestingly, the use of herbal remedies to treat livestock ailments is still alive in the Dugda District. The result showed that in the District, farmers and traditional healers are rich in ethnoveterinary knowledge and utilize medicinal plants to treat livestock ailments. This may be related to the inhabitants’ strong relationship with livestock, its location between Arsi and Gurage Highlands. It could be also attributed to familiarity of local people to ethnoveterinary remedies, which make them feel safe in using traditional remedies. In addition, movement with livestock in search of grazing land (*Godansa*) and *Gada* grade celebrations (*Chaffe Godansa*), where they fetch law governing everything including livestock, human being and natural resources. Such activities might have helped them to keep the traditional livestock healing culture and share among themselves and beyond. And the presence of medicinal plants (64) indicated the potential of the District as an ethnoveterinary resource pool, despite the high rate of degraded habitats that continued for so long, its semi-arid nature and drought prone. Similar ethnoveterinary studies elsewhere in Ethiopia also reported comparable number of medicinal plants, to mention a few, 43 medicinal plants [17]; 46 [15]; 49 [18]; 51 [19]; and 53 [29]. Some of the studies documented higher number of ethnoveterinary medicinal plants than the current results, such as by Yineger et al. [16] (74 species), Yigezu et al. [33] (74) and Dinbiso et al. [54] (103), at Bale Mountains National Park, four districts of Jima Zone and Dawuro Zone of Ethiopia, respectively. Thus, the present and early ethnoveterinary inventories showed the importance of medicinal plants in management of different livestock ailments, particularly, in areas where modern veterinary services are absent, expensive, inaccessible and scanty [15]. This could be also related to poverty, the availability of medicinal plants, better accessibility and low cost of herbal medications. However, ethnoveterinary practices are vary from place to place and that is related to the diverse culture and tradition of the people as well as the vegetation type of a particular area [15].

The analysis of botanical families of ethnoveterinary plants revealed that Fabaceae, Apocynaceae, Cucurbitaceae, Solanaceae, Malvaceae and Vitaceae families are the dominant families in ethnoveterinary medication systems of Dugda District. This finding is in line with other similar ethnoveterinary studies conducted elsewhere in Ethiopia [15, 18, 19, 27, 29, 31, 33], where these families were reported as important in their respective ethnoveterinary services. The greater number of species in these families could be attributed to their wide distribution and abundance in the *Acacia* wooded grassland of the Rift Valley (ACB/RV) vegetation, the study area in particular. The member species of these families have developed drought-resistant mechanisms to dwell in semi-arid dry land of Great Rift Valley of Ethiopia. Besides, Fabaceae, Cucurbitaceae, Solanaceae and Apocynaceae families are also among the top 25 families of vascular plants of Ethiopian flora area [55] and most frequently utilized plant families to treat livestock ailments in the country [21]. Thus, the preference of members of these families could be related to availability in close vicinity, presence of bioactive ingredients in member of these families and long-term familiarity with these herbal materials [56].

In the study area, various types of medicinal plants’ life forms are employed, among which herbs accounted the highest share, followed by shrubs. The dominance of herbaceous species could be related to their rapid grow during bimodal rainy seasons of the area, availability around homestead and ease of collection. As described in Giday and Teklehaymanot [18], shrubby species are also better adapted to arid conditions as compared to plants of other life forms, which made them abundant and available in such areas for farmers and pastoralists in need. Frequent use of herbs in ethnoveterinary medications were reported in ethnoveterinary studies investigated in different part of Ethiopia [15, 16, 32, 54, 57], similarly others recorded shrubs as important herbal medications [18, 19, 27, 28]. In contrast to present finding, tree species constituted the largest share in other ethnoveterinary studies [22, 29, 33]. This similarity and variation in utilization of life forms of ethnoveterinary plants in different communities could be attributed to agro-ecological settings, indigenous knowledge exchange and independent development of indigenous knowledge in their respective communities.

### Plant parts used for remedy preparation

The other important attributes of ethnoveterinary plants analysed were plant parts used for preparation of veterinary remedies. The study revealed that leaves are the most sought plant part for remedies preparation followed by roots. This collection of leaves as major ethnoveterinary treatments could be related to high phytochemical constituents, ease of collection and preparation of remedies, and readily availability in time of need. In agreement with current result, leaves have been reported as commonly utilized plant parts in ethnoveterinary medical systems of different ethnic groups of Ethiopia [15, 18, 22, 28, 31, 33, 54, 58] and elsewhere in the world [25, 59–63]. These findings differ from the results of Yineger et al. [16] who reported frequent use of roots in ethnoveterinary medical system of communities living around Bale Mountains National Park of Ethiopia. Interestingly, use of leaves as a single remedy or concoction with other parts in the study area is important for sustainable utilization of medicinal resources, because leaves are renewable resources of medicinal plants in contrary to the roots. Several studies [18, 59] indicated that gathering leaves are recommendable, as its collection does not result the death of the entire plant. However, conservation issues have been raised by other studies [19, 63], due to ethnobotanical collection of roots and whole parts, which may be destructive and unsustainable, as harvesting of roots may increase the risk of the medicinal plants extinction.

Ethnoveterinary remedy of Dugda District is largely prepared from freshly harvested plant parts. Preference of freshly made remedies could be attributed to attaining volatile essential oils and secondary metabolites, which are important to fight disease-causing agents in livestock with high efficacy and curative power, these bioactive ingredients will escape or be degraded upon drying. In line with this, traditions of using ethnoveterinary remedies prepared from fresh plant materials were widely observed in different cultural communities of Ethiopia, for instance, in ethnoveterinary medication of Afar people of Ada’ar District [18], Ankober District [19] and Mojana District [27].

### Methods of remedy preparation and routes of application

In remedy preparation, local people and traditional healers strictly follow ways needed to get the intended livestock remedies. The current findings showed that pounding is the most common mode of ethnoveterinary remedies preparation, and closely followed by grinding. According to Gakuubi and Wanzala [73], modes of recipe preparation largely depended on the type of targeted medicinal plant, parts of plants employed, type of disease and the livestock types being treated. And also it could be related to long-term experiences of traditional healers on modes of remedy preparation and application with best preforming ethnoveterinary regimens [19].

Furthermore, local communities also took into consideration the number of medicinal plants involved while preparing herbal remedies based on ailments and livestock types. In this particular study, large portion of livestock remedies are formulated from single medicinal plants. The use of single plant species for the majority of ethnoveterinary recipes were also common in different cultural groups of Ethiopia [15, 18, 19, 31, 54] and other countries [3, 60, 62, 64–66]. As Grade et al. [66] stated, the mono-preparations of veterinary remedies could be related to confidence, experiences and deep ethnoveterinary knowledge of farmers and traditional healers to select only one specific remedy. In contrast, the dominance of poly-herbal preparations were also documented in other ethno-lingual communities, such as at Bale Mountains National Park [16]; four Districts of Jimma Zone of Ethiopia [33]; and in Buuri district of Kenya [63]. These authors argue that concoction of two or more plants would increase synergistic effects (effectiveness), neutralize toxicity effects and/or bitterness, palatability and ease of application of ethnoveterinary remedies. Furthermore, the use of ethnoveterinary medicinal plants is also shaped by familiarity of local communities with their environs, vegetation types, seasonality and ease of availability of herbal material in that particular area [56].

Regarding solvents used in livestock remedy preparations, cold water was the most principal solvent for remedy extraction. Similar observations were also made by ethnoveterinary studies in Ethiopia [19, 28, 31, 33, 54] and in pastoral Karamoja of Uganda [66], where cold water was preferred as best and effective solvent in extraction of active ingredient found in medicinal plant used in management of livestock diseases. However, other findings elsewhere in the world [59, 61, 67, 68] revealed that ethnoformulation of decoctions in boiling water was the prominent methods of ethnoveterinary recipes preparation. Maphosa and Masika [23] further explained that boiling plant material in water for a long time to form a decoctions may promote extraction of water soluble polar compounds or it could detoxify harmful substances, but the method is generalized one and less carefully selected plant parts are used [67]. In addition to herbal materials and water, non-plant materials such as salts, milk, butter, etc. are also used as important ingredients in ethnoveterinary system of the study area. These substances enhance ethnoveterinary remedies to be dissolved, improve its palatability and medicinal properties and as a vehicle system, during intake or topical application of remedies. Incorporating non-plant material in ethnoveterinary medicine preparation is not exceptional to the study area, while it is common practices in different ethno-lingual communities of Ethiopia [18, 33] and other parts of the world [62, 63, 65, 66]. In the study area, some special mode of remedy preparation and application was also observed, for example, simple surgical method on dewlap of oxen with swollen hump, where traditional healer slice root of *Kalanchoe petitiana* tie with string, then insert into dewlap to drawdown pus accumulated in hump.

In addition, non-plant veterinary remedies have also been documented in the study area, which are used to treat livestock ailments, these include: mixture of charcoal, burned petrol and water (for bloating), burned petrol (for wound), ash (for wound, bone fracture or dislocation), limestone (for emaciation and ectoparasites), salt (for eye disease), urine (for bloating), edible oil (bloating), honey (for gastrointestinal disorders), dough and whey (emaciation), faeces (for wound), cattle skin (for FMD), muck (smoking for BEF), sacrifice of black female sheep or goat (for evil spirit or evil eye), spleen (for anthrax) and hot iron (for blackleg and abscess). Non-plant ethnoveterinary remedies were also recorded by similar ethnoveterinary inventories conducted elsewhere in the world [13, 26, 65, 68, 69]. These uses of non-plant remedies for the management of livestock ailments could be better alternative for conservation of wild medicinal plants that are already under great threats.

As regards to sources of medicinal plants, the majority of therapeutic plants were collected from the wild. The dominance of wild medicinal plants in ethnoveterinary medicines could be related to the perceptions of indigenous people that wild plants are more potent than cultivated plants [26]. In conservation point of view, the predominance of wild sources point out presence of high impact on wild ethnoveterinary medicinal plants of the area due to ethnoveterinary collections [16, 19] and little practices of medicinal plant cultivation, which in long term diminish these vital plant resources. Ethnobotanical collection of medicinal plants from wild habitats for their roots put some plants under great pressure, like *Withania somnifera*, *Phytolacca dodecandra*, *Cucumis ficifolius*, *Cyphostemma pannosum* and *Gomphocarpus fruticosus*, as these plants are rarely found in the study area. Similarly, dependency of indigenous people on wild medicinal plant for their ethnoveterinary practices has been widely recorded by various ethnoveterinary studies in Ethiopia [15, 16, 18, 19, 49, 54] and elsewhere in the world [26, 62, 68]. As alternative to wild sources, studies advocated cultivation and conservation of medicinal plants as better strategy for livestock health and food security, overall socio-economic development of rural poor populations [54].

Oral routes administration of remedies in the form of drenching are the most frequent route of application used to treat livestock ailments. This could be related to prevalence of diseases affecting internal organs, where this route assists rapid physiological reaction with the intended disease-causing pathogens and increasing effectiveness of the medicines. Concur with present study, bulk of ethnobotanical studies have recorded oral application as prominent route in Ethiopia and other parts of the world [18, 29, 33, 54, 59, 61, 65]. The current findings are in different to those obtained by Yirga et al. [70], who found dermal application as commonly used route of administration in ethnoveterinary medications of Seharti-Samre district, Northern Ethiopia. As the case in the present study, lack of standardized dosage for ethnoveterinary regimens were also documented in ethnoveterinary practices of different ethno-lingual communities elsewhere in the world [18, 19, 60, 71, 72], the situation that made veterinarians hesitate to use ethnoveterinary medicines.

### Widely used medicinal plants and types of livestock treated

The use reports analysis showed that some ethnoveterinary medicinal plants are most widely used in ethnoveterinary services than others in the study area. As a result, *Withania somnifera*, *Kedrostis foetidissima*, *Cyphostemma cyphopetalum*, *Gymnanthemum amygdalinum*, *Calpurnia aurea*, *Cucumis ficifolius*, *Senna occidentalis*, *Phytolacca dodecandra* and *Croton macrostachyus* are among the wildly used medicinal plants in the study District. According to Aziz et al. [56], wide acceptance of certain medicinal plants in ethnoveterinary medicines may be due to their efficient activity, large availability in the area and long history of use in traditional medicine, the situations that make them more feasible to use than plants that are difficult to harvest. On the other hand, the most frequently mentioned medicinal plants, it does not always mean they are more effective to treat ailments than less frequently cited medicinal plants. Because some ethnoveterinary knowledge of medicinal plants are common and shared while others are more localized and specific to certain communities, family lines and even individuals [7]. That means, medicinal plants with low use reports may be found in custodies of a few specialist traditional healers [7], for that matter such medicinal plants need future attentions. In this particular study, for example, ethnoveterinary knowledge related to medicinal plants used to treat rabies are very secret, held by a few individuals in the community. In addition, low use reports of medicinal plants also indicate indigenous knowledge associated with them are under risk to be lost.

The majority of ethnoveterinary plants are employed for the treatment of cattle ailments, while a few medicinal plants are used to treat goats and sheep, all livestock, equines, dogs, cats and poultry. This could be attributed to high prevalence of cattle ailments and cattle dominance in number in the study area and/or very strong cultural and economic attachments between cattle and indigenous people. Similar observations were made in other socio-cultural groups of Ethiopia, for examples, most ethnoveterinary remedies used to treat cattle in Ada’ar District of Afar region [18]; Ankober District of Amhara region [19]; and Dawuro Zone [54] of Ethiopia, which indicate better alternative to improve the welfare of cattle in the country. However, in ethnoveterinary practices of Abergelle, Sekota and Lalibela districts of Amhara region of Ethiopia, most veterinary recipes were being used to manage all domestic animals [29]. Such ethnoveterinary practices might be related to presence of fair ethnoveterinary knowledge associated with all livestock types and other domestic animals for management of their respective ailments, as compared to areas with high tendency to veterinary remedies used to treat cattle diseases.

### Ethnoveterinary ailment category and fidelity level of medicinal plants

The informants’ consensus factor (ICF) for each ailment category is found between the ranges of (0.69–0.94). The results indicated that there is a well-defined selection criterion in the study community and informants have great agreement on indigenous and local knowledge of ethnoveterinary plants and practices [73]. It also determines the cultural consistency of the selection of a set of medicinal plants used in the treatment of a certain illness category [39]. The highest ICF values indicate high incidence of the disease category in the area. As well as, medicinal plants used to treat ailment categories are probably with high potency and important to select potential candidate medicinal plants for phytochemical and pharmacological research.

Furthermore, gastrointestinal diseases are reported to be treated by large number of medicinal plants as well as with high number of citations. Dermatological diseases are also treated by good number of therapeutic plants. This might be due to high prevalence of these disease categories in the study area. It also indicated that local people share the knowledge of the most important medicinal plants to treat the most frequently encountered livestock ailments in the area. Similar ethnoveterinary survey carried out in Ankober District of Ethiopia also recorded gastrointestinal, ecto- and endo-parasitic and dermatological as major and prevalent ailment categories of the area [19]. Berhanu et al. [57], similarly, reported that gastrointestinal, dermatological and febrile disorders are ailment categories with high frequency in Ambo District of Ethiopia. In addition, as botanical surveys in different part of the world indicate gastrointestinal disorders are the most frequently reported and prevalent ailment category throughout the world [56, 59, 64, 65, 73].

Medicinal plants with high fidelity-level value (> 50%) in each ailment category showed that these plants are abundantly found in the area and frequently used by local communities in the treatment of livestock ailments. As explained in Friedman et al. [74], if FL > 50%, then there is high degree of consensus around the use of this species for treatment of that particular ailment category, which makes plant appropriate candidate to treat this type of disease. It also shows relatively high healing potential of the species for treating ailments under the respective ailment categories and good candidate for further pharmacological studies [75]. However, some medicinal plants including *Cyphostemma cyphopetalum, Withania somnifera, Gymnanthemum amygdalina* and *Cucumis ficifolius* had low FL values in their respective disease categories. The low FL values show the local inhabitants disagree on use of medicinal plants to treat particular ailment categories, it could be due their multipurpose in ethnoveterinary medicine formulation.

### Relative importance of ethnoveterinary plants

In Dugda District, 27 (42.19%) medicinal plants were reported to be multipurpose species with diversity of remedies ranges (2–8 remedies). In this regard, the most multipurpose medicinal plant species in the study area were *Croton macrostachyus*, *Kedrostis foetidissima, Withania somnifera*, *Senna occidentalis* and *Gymnanthemum amygdalinum*. For instance, *C. macrostachyus* was used to treat eight specific ailments and five disease categories including gastrointestinal, dermatological, musculoskeletal, neurological and general illness. Similar observation was made by Dinbiso et al. [54] and Lulekal et al. [19] in Dawuro Zone and Ankober District of Ethiopia, where *C. macrostachyus* is used for treatment of multiple veterinary ailments, 10 and 8 ailments, respectively. Multipurpose of *W. somnifera* and *G. amygdalinum* are also documented in ethnoveterinary practices of four District of Jimma zone of Ethiopia [33]. Similarly, *S. occidentalis* is also a multipurpose species in other part of Africa [66, 76]. According to Giday and Teklehaymanot [18], medicinal plants with high relative importance (RI) indicate their relative abundance in that particular area. Furthermore, these plants are the most widely utilized ethnoveterinary medicinal plants in Ethiopian ethnoveterinary medicines [15, 19, 28, 29, 32]. Multipurpose of these medicinal plants could be attributed to their phytochemical constituents with wide spectrum bioactivity against wide range of ailments. Of the nine ailment categories, gastrointestinal ailments were reported to be treated by high number of medicinal plants, and closely followed by dermatological and general illness. In support of these results, Bennett and Prance [52] explained that gastrointestinal, skin and respiratory remedies are the most frequently reported types of medicines throughout the world.

### Distribution of ethnoveterinary medicinal plant knowledge

In the present study, the comparison of ethnoveterinary medicinal plant knowledge showed significant difference, where key informants cited more medicinal plants than general informants. This could be attributed to the secret practice and transfer of ethnoveterinary knowledge along the family line, to safeguard their benefits from the knowledge and fear of loses of healing power of plant when disclosed. And it could be also related to long-term experience of key informants in using medicinal plants to treat livestock ailments. These findings in line with previous ethnoveterinary researches in Ethiopia [27, 28] that showed significant difference between key and general informants in ethnoveterinary plant knowledge. There was no significant difference observed in medicinal plant mentioned by male and female informants. A similar observations were made in another ethnoveterinary inventories in Ethiopia [27, 54] and differ from that of Asfaw et al. [28]. The comparison of medicinal plants cited by the two age groups (20–39 years and 40–87 years) showed significance difference. This indicates matured groups are more knowledgeable than young groups in using medicinal plants for veterinary uses, which was also reported from the early work [19], but dissimilar with that of [54]. It might be related to high experience of elders, close connection to natural ecosystem and livestock, faith on medicinal plant healing power and also knowledge gaps between generations. Similarly, the number of medicinal plants cited by illiterate and literate informants showed significant difference. This disparity could be attributed to the reliance of illiterate communities on medicinal plants to manage livestock ailments, while educated people prefer other options mostly modern drugs. It might be also related to poor connection of educated people with the traditional societies and natural ecosystems. Similar finding has been reported in ethnobotanical researches elsewhere in Ethiopia [19, 27]. In conclusion, ethnoveterinary medicinal plants knowledge is being threatened by globalization, expansion of modern education and ways of the knowledge transfer.

### Importance of the current study in animal health and food security

Animal health is the important concern of ethnoveterinary study, because livestock are one of the pillar on which the livelihood of the communities rests. In the study area, the most prevalent ailments are anthrax, inappetence, diarrhoea, rabies, bloating, blackleg and mange. The aforementioned ailments are also the commonly encountered livestock ailments elsewhere in Ethiopia, which can cause substantial losses in livestock and lower productivity [14, 21]. The people in the study District and majority of livestock raisers elsewhere in Ethiopia have limited access to modern animal health services to deal with the encountered livestock ailments [77]. This is because local communities are geographically far from the sites of veterinary stations and those that are closer to the sites may not afford the fees for services. In such areas, farmers and pastoralists partly or solely depend on ethnoveterinary medicinal plants to sustain animal health. As described by Silva et al. [78], local people intentional or unintentional use therapeutic plants as ethnoveterinary medicine for food-producing animals that could improve the well-being and quality of derived-food products. This makes ethnoveterinary medicines a good alternative to modern drugs that used to manage livestock health in general and food-producing animals, in particular [78]. Particularly, its significance is paramount in arid and semi-arid areas, as livestock provide major food security in times of crop failure [10]. Because livestock tend to be more resilient than crops when disasters such as drought and floods strike [79]. Thus, ethnoveterinary medicinal plants could play a vital role in such areas to improve livestock health, which in turn used to ensure household food security and income generation options, and contribute to national livestock economy. Moreover, medicinal plants has the potential to improve the economic status of local communities and in alleviating poverty through marketing and long-term use [54].

When using ethnoveterinary medicine, we have to be curious about its dosage, since dosage is a determining factor, in small quantities as medicinal and large amounts may leads to toxicity or lethal to animals. In study area, some reported ethnoveterinary medicinal plants are with toxic potential (e.g. *Croton macrostachyus*, *Melia azedarach*, *Xanthium spinosum, Calotropis procera*, *Datura stramonium* and *Phytolacca dodecandra*). The ethnoveterinary practices of these medicinal plants could be poison due to substance accumulation (residues) or overdose [78]. The ethnoveterinary practices of toxic plants can be harmful to livestock, risky to human health (a concern on food safety) and may lead to harmful economic consequences [78]. Therefore, as stated by Silva et al. [78], shortcomings of ethnoveterinary practices are toxicity/inefficacy and lack of standardization that should be validated through pharmacological properties studies with toxicity investigations.

Furthermore, some prevalent livestock ailments in the study area are zoonotic diseases (anthrax and rabies), which are treated by medicinal plants. Anthrax and rabies are among the five prioritized zoonotic diseases that Ethiopia has committed to control, using a One Health approach [10]. Thus, current findings also offer crucial insight for One Health approach in controlling zoonotic diseases in Ethiopia and beyond and also illustrate the importance of ethnoveterinary medicinal plants in both livestock and human health problems.

### Comparison with previous studies in Ethiopia

Comparison of current study with the ethnoveterinary studies in Ethiopia showed that 56 medicinal plants are documented in various similar studies in the country to treat different livestock ailments. The most frequently documented medicinal plants include *Achyranthes aspera*, *Calpurnia aurea*, *Croton macrostachyus*, *Cucumis ficifolius*, *Datura stramonium*, *Phytolacca dodecandra*, *Solanum incanum*, *Verbascum sinaiticum* and *Gymnanthemum amygdalinum* (Table 8). Tihahu et al. [21] also reported the frequent utilization of these ethnoveterinary medicinal plants in the country. According to Lulekal et al. [19], similarity in use of ethnoveterinary medicinal plants in different communities could be attributed to cross-cultural exchange of indigenous knowledge among different ethnic groups, within similar ethnic group and also availability of the species in use in those areas. This could be also related to similarity in climatic and weather conditions, geographical location and communities’ faith on medicinal plants healing power [21]. In addition, their frequent utilization might be coincide with the presence of bioactive ingredients against livestock ailments [21], as evidenced in various pharmacological studies and shown promising antimicrobial activities [80].

**Table 8:** Comparison of reported ethnoveterinary medicinal plants with previous studies in Ethiopia

Medicinal plants and CS	Ailments treated in Dugda District	Ailments recorded in previous study in different regions of Ethiopia treated by recorded medicinal plants					
		AmR	AfR	OR	SR	SNNPR	TR
*Achyranthes aspera*	Diarrhoea, equine diseases, mange/alopecia and blackleg	Nasal infection [19], eye infection [19, 29, 86], bleeding [19, 87], leech and wound [28], bloat [88], bone fracture [87, 89], blood clotting [90, 91]	–	Blackleg [13], bleeding [32], glanders [16], abdominal disorder, endoparasite and febrile illness [92], mastitis [93], wound [93, 94]	–	Sudden sickness, cough and skin cancer [95], ectoparasite [96]	Mastitis, wound and diarrhoea [97], anthrax and babesia [98], snakebite and wound [99], eye infection [98, 100–102], rectal prolapse [70], joint dislocation [103]
*Allium sativum*	Anthrax and blackleg	Blackleg [19, 28], dermatophilosis, mange, scabies, ringworm, leech and lice in chicken [19], anthrax [28], pasteurellosis [29], bloat and coccidiosis [89], helminthiasis [19, 89], tryps [91], general illness [27]	PPR [104]	Stomachache and abdominal pain [57, 93, 94, 105], cough, fungal and leech [106], colic and bloat [57, 107], wound [32], expectorant and antiseptic [17], hepatitis [16], pasteurellosis and boat [93], mastitis, diarrhoea, endoparasites [30, 107], septicemia [30]	–	Blackleg [15, 54], babesiosis [54], leech [108]	Horn worm and leech [100], eye disease, aspergillosis, FMD, Newcastle disease [102]
*Aloe trichosantha* (LC)	Mange/alopecia, LSD, wound and hump swelling	–	Anthrax, CCPP and CBPP [18], blackleg, joint illness and brucellosis [104]	Anthrax [36]	CCPP/cough [109]	Swelling [110]	–
*Asparagus africanus*	Wound and rabies	Coccidiosis [19], swelling [29], gastrointestinal ailments, bone fracture, mastitis and poor mothering [89]	–	Rabies [16], anuria, heart failure and wound [92], glanders [36]	–	Blackleg, pneumonia and bloat [30], cow disease [111], wound [112]	Body swelling [100], joint dislocation [113]
*Balanites aegyptiaca* (LC)	Anthrax and blackleg	–	Anthrax [18, 114], Render pest [114], CCPP, tryps [18, 104], bloat, colic, sudden sickness and diarrhoea [18], brucellosis, blackleg, pasteurellosis and BTB [104]	–	Diarrhoea [109]	Eye infection [110]	Abdominal problems and BTB [115]
*Calotropis procera* (LC)	Wart, ringworm and blackleg	Mange [19], tumour [28], caseous lymphadenitis [29], swelling [88], blackleg [89]	Blackleg [18, 104, 114], colic and prophylaxis [18], anthrax, mastitis and contagious agalactia [104]	Blackleg [36]	–	–	Swelling [98, 99, 115], wound [99, 115], actinobacillosis [70], sore [102], warts [115]
*Calpurnia aurea* (LC)	Anthrax, ticks and lice infestation, rabies and prophylaxis against rabies	Ectoparasites: tick and lice [19, 27, 28, 87, 89], helminthiasis, sore and parasitic leech [19], snake bite [19, 116], rabies, anthrax, coccidiosis and pneumonia [89], diarrhoea [87, 90, 116]		Ectoparasites: pediculosis and lice [30, 33, 57, 93, 94, 117–119], rabies [107, 117], bloat [32, 93], blackleg [33, 118], respiratory ailments and diarrhoea [33], dermatophytes and scabies [16, 33, 105, 107, 120, 121], leech and abdominal pain [93], anthrax, hepatitis and earache [118], snake bite [32, 121], endoparasite [30, 94]	Endoparasite [109]	Ectoparasites: tick, lice, flea and louse [54, 108, 110, 122], mastitis [123], snake bite [124], eye infection, urine retention, blackleg and anthrax [112]	Ectoparasites: ticks, lice and fleas [97, 98], mastitis and dermatophilosis [97, 99, 100], mange [101, 115], Salmonellosis, *E. coli* infection, lichen simplex chronicus and sheep pox [102]
*Capparis tomentosa*	Fattening and strengthening	Caseous lymphadenitis [29], 2018), rabies [89], evil spirit [86]	–	Skin disease [36]	–	–	–
*Capsicum frutescens* (LC)	When cow refuse its calf	–	Pasteurellosis and flue [104]	–	–	Kidney problem and grain overload [54], cattle disease [111]	Bloat [100]
*Carissa spinarum* (LC)	Rabies	Helminthiasis and parasitic leech [19], lung disease [28], eye treatment [29], evil eye, wound and diarrhoea [116], gastrointestinal problem, rabies and anthrax [89]	–	Breathing problem [57], urinary tract infection [32], helminthiasis [17], ringworm [17, 93], febrile illness and evil eye [92], wound [93], rabies [107], evil spirit and dermatitis [125]	–	Increase milk production [15]	–
*Cissus quadrangularis*	Anthrax	–	Anthrax [114], blackleg [18], swelling [104]	Blackleg [118]	Tick, wound, mastitis, helminthiasis, leech and blackleg [31]	Evil eye [122]	Asthma [103]
*Crinum abyssinicum*	Rabies	–	–	Endoparasites [94]			
*Croton macrostachyus* (LC)	Bloat, diarrhoea, inappetence, bone fracture, dullness, blackleg, anthrax, BEF and LSD	Dermatophilosis/mange, scabies, bleeding and sore [19], foot rot [27, 28], blackleg [29], rabies [89, 116], coccidiosis and helminthiasis [89], bloat [87, 89], ringworm [19, 90, 126], rabies [90], trauma [27]	–	Ringworm ([13, 17, 93, 105], wound [93, 105, 117, 118], skin diseases: lesion and scabies [30, 33, 105], evil eye [105], febrile illness [22, 92, 105], blackleg [33, 117], lymphangitis [57], foot rot [32, 57], bloat [13, 17, 32, 33, 94], respiratory ailments and lung diseases [22, 94, 118], rabies [30, 107], ASH and diarrhoea [33], anthrax [22], abdominal pain/colic [92, 107], toothache, swelling, tetanus, abortion and hepatitis [118], anthelmintics [127], mastitis, tryps and septicemia [107], typhoid and liver [94], rectum prolapse and ectoparasites [30]	–	Blackleg [15, 54, 108, 110], constipation [15], equine colic [128], abdominal pain and stomachache [30, 111, 128], bloat [30, 128], diarrhoea [30, 111], ectoparasites [30], cancer [129], endoparasites and Bovine/ovine pasteurellosis/lung worm and wound [54], fungal [111], sudden illness and indigestion [110]	Leech [70], anthrax and bloat [103], scabies [102], splenomegaly, Ovine pasteurellosis and rabies [115]
*Cucumis ficifolius*	Bloat, blackleg, diarrhoea, mineral deficiency and mange/alopecia, anthrax, BEF and LSD	Blackleg [27, 28, 88], rabies and rabies vaccine [28, 88], anthrax [89, 90], diarrhoea [87, 89], gastrointestinal ailments [89, 91], coccidiosis, cowdriosis, hepatitis and wound [19], rurt and foot rot [28], tryps and cough [88], abortion, phimosis and septicemia [89], tryps [91], liver fluke [130]	–	Blackleg [16, 22, 33, 107, 119], rabies [93, 125], cattle infection [105, 121], endoparasite [32, 57, 94], retained placenta [32], colic and emaciation [107], bloat and blood clot [94]	–	Blackleg [108], eye problem [95], bloat, anthrax and tryps [110]	Anthrax and abdominal pain [98, 99], infection and Hyena bite [102]
*Cynoglossum lanceolatum*	Inappetence	–	–	Mastitis and blackleg [107]	–	–	Wound [100]
*Cyphostemma adenocaule*	BEF, inappetence, anthrax, lymphangitis and blackleg	Blackleg [29], bloat and leech [89]	–	Blackleg [120]	–	Mastitis, evil eye, dermatophilosis, LSD and FMD [54], colic, leech and snake bite [108]	Wound [97], snake bite [98, 99], pack sore [102]
*Cyphostemma cyphopetalum*	Anthrax, bloat, colic, rabies, prophylaxis against rabies, diarrhoea, blackleg, BEF and LSD	Swelling [29, 89], gastrointestinal ailments [89], rabies [91]	–	–	Wound [31]	Wound [110]	–
*Cyphostemma pannosum*	BEF, equine diseases, mange/alopecia and colic	Rabies, myiasis, foot rot, hyena bite and equine saddle sore [89]	–	–	–	Anthrax, pasteurellosis and fattening [108]	–
*Datura stramonium*	Rabies and wound	Swelling [29], tryps [88], mastitis [89], myiasis [27]	Nerve problem [18]	Wound [105], systemic illness [57], ringworm [32], blackleg and respiratory ailments [33], coenurosis [22], rabies [118], evil eye [94]	–	Coughing in equines [122], toothache [131], listeriosis [54], rabies, leech, gum problem and ringworm [108], worm infestation [111], wound [96, 110], bloat [110]	Anthrax [98, 99], wound [99], diarrhoea and shivering [100], blackleg and nasal bleeding [103], mange [115]
*Dodonaea viscosa* subsp. *angustifolia*	Mange/alopecia, blackleg, anthrax, BEF and LSD	Bloat, diarrhoea, ringworm and scabies [19], leech, hepatitis and rheumatic [116], bone fracture [87]	–	Wound [32, 105, 120], bone fracture [32, 57], diarrhoea [22, 33], bloat and live disease [33], leech [93], ectoparasites [118], anthelmintic/tapeworm [105, 127]		Scabies [15], anthrax, cancer, sun stroke and stabbing pain [129], endoparasite, diarrhoea and sudden sickness [95], ectoparasites [110, 112], lymphatic swelling [95, 112], bloat and fattening [110]	Wound [97], dislocated bone, body part [98, 99, 102], sore [102]
*Echinops hispidus*	Inappetence	–	–	–	–	Bloat and fattening [110]	–
*Foeniculum vulgare*	Mange/Alopecia, urine retention/urolithiasis, blackleg, anthrax, BEF and LSD	–	–	Abscess [22], to remove plastic materials from livestock stomach [118]	–	Bloat [110]	Urine retention and abdominal pain [100]
*Gomphocarpus fruticosus*	Ringworm, FMD, scabies and wart	Bloat, poor appetite and sudden diarrhoea [19]	–	Diarrhoea and lung disease [16]	–	–	–
*Gossypium hirsutum* (VU)	Eye disease	–	–	–	–	Blackleg [108]	–
*Grewia ferruginea*	Retained placenta	Retained placenta [87, 89]		Rabies [22], retained placenta [107, 121]		Cough [112], retained placenta [110]	Leech [103, 115], retained placenta [115]
*Gymnanthemum amygdalinum*	Evil spirit, diarrhoea, bloat, colic, mange/alopecia, blackleg, anthrax, BEF and LSD	Retained placenta and CBPP [19], jaundice [28], blackleg, mange mites, tania and pasteurellosis [27], bloat and urinary problems [87, 89, 116]	–	Endoparasite [13, 33, 57, 117, 127], retained placenta [33, 93, 117, 119], febrile illness [57, 92], bloat [57, 118], diarrhoea [13, 32, 33, 94], blackleg, respiratory ailments and to improve milk production in cows [33], hepatitis [16], urine retention [118]	Endoparasite [109]	Tryps, its prevention and tsetse fly control [15, 54], diarrhoea [30, 95, 110], improve milk production [15], LSD and skin problem [30, 128], pasteurellosis and endoparasite [128], cough and stabbing pain [129], anthrax, sudden illness and prevent contagious disease [110]	Bloat [100]
*Hypoestes forskaolii*	Colic in equine	–	–	–	–	Increase milk production [110]	Babesia [98], anthrax [99]
*Ipomoea carnea*	Rabies	–	–	Diarrhoea [57]	–	–	–
*Kalanchoe petitiana*	Hump swelling	Swelling and anthrax [88], fascioliasis [89]	–	Swelling/abscess [13, 16, 120], nasal bleeding [105], endoparasite [117], constipation [16], eye disease [107]	–	–	–
*Kedrostis foetidissima*	Diarrhoea, bloat, colic, fattening and strengthening, BEF, lice, retained placenta, lymphangitis and mange/alopecia	–	–	Constipation, rough hair coat and urine retention [36]	–	–	–
*Lagenaria siceraria*	Rabies and many disease conditions	Rabies [126]	–	Blackleg and retained placenta [117], rabies and tryps [107]	–	–	–
*Linum usitatissimum*	Retained placenta	Retained placenta [89, 116]	–	Retained placenta [32, 57, 107, 121], constipation [32, 57], gastritis [32], dandruff [119]	–	Emaciation/fattening/bone broken, constipation [54], retained placenta [111]	Prolonged delivery [100], retained placenta [102]
*Melia azedarach* (LC)	Colic, bloat, diarrhoea, inappetence and chicken diseases	Coccidiosis [89], insect repellent [86]	–	Diarrhoea in chicken and cattle [120], against various disease conditions [13], anthelmintics [127]	–	–	Wound [98, 99], ectoparasite like lice [101, 102], bloat [100], mange mites [70]
*Momordica foetida*	Bloat	Leech [29], swelling [88, 89], blackleg [88], wound and febrile illness [116], ascariasis [89], sun stroke [87]	–	Blackleg [13, 33], colic and endoparasite [33], anthrax and febrile illness [22], metritis for good flavour [92], fracture, rabies, tryps and myiasis [107], ectoparasites and warts [94], babesiosis and anaplasmosis [30]	–	Blackleg [15, 54, 122], anthrax [15, 108], diarrhoea and amoebiasis [19, 54, 110], bloat [54], rabies and leech [108], evil spirit and snake bite [110]	–
*Nicotiana tabacum*	Coughing and nasal discharge	Blackleg [28, 116], leech [27, 28, 86, 89, 116, 130], diarrhoea [29], cough [116, 126], coccidiosis [89]	–	Bloat [32, 57, 105], leech [30, 32, 57, 93, 94, 105, 119, 121], endoparasites: tapeworm [93, 105, 107, 121, 127], blackleg [16, 33, 107], trypanosomiasis [105, 121], snake bite, snake poison and snake repellent [33, 92, 94, 107, 117, 119], eye infection [105], abdominal pain and colic [57], diarrhoea, respiratory ailments and for fattening of cattle [33], ectoparasites: tick and lice [30, 94, 107]	Body swelling [109]	Ectoparasites: tick [30, 108, 110], leech [15, 30, 110], blackleg [54, 108], wound [131], bovine/ovine pasteurellosis/lung worm [54], stomachache [95, 108], bloat, cough and fattening [110]	Leech [97–102], scabies and lice infestation [98, 99], plant toxin/toxicosis and tryps [102]
*Ocimum gratissimum* subsp. *gratissimum*	Anthrax	Eye treatment [29]	–	Febrile illness [57]	–	Retained placenta [110]	–
*Opuntia ficus-indica* (DD)	Emaciation, diarrhoea and mange/alopecia	–	–	Blackleg, sudden sickness and febrile illness [92]	–	–	Anthrax and lice or fleas [99]
*Phytolacca dodecandra*	Ringworm, rabies, mandibular oedema, eye disease, FMD, BEF, constipation and prophylaxis against rabies	Leech [19, 27, 87], rabies [28, 29, 87, 89], helminthiasis: [19, 89], extoparasites: lice in chicken and coccidiosis [19, 87, 89], mange and LSD [19], rabies [28, 29], blackleg [27], eye disease [89, 116], anthrax [89, 130], bloat and ascariasis [89], ringworm [90], constipation [91]	–	Rabies [57, 93, 94], endoparasite/anthelmintics [93, 127], lymphangitis [120], liver disease [105], eye disease [94, 107, 117], leech [57], glanders [16], coughing in equines [118]	–	Blackleg [15, 54], leech [54, 110], rabies vaccine [110], wound, cancer/tumour, calf diarrhoea, constipation and endoparasite [128], diarrhoea and difficult urination [30], bovine/ovine pasteurellosis/lung worm/cough [54, 110], stomach problem and sudden sickness [95]	Rabies [101, 102, 115], eye infection [101], ectoparasite [101, 102], leech [100, 115], scabies [102], blackleg [103], anthrax [101, 103], warts [115]
*Pterolobium stellatum*	Evil eye	–	–	Evil eye [105]	–	–	Bone dislocation [98, 99]
*Searsia natalensis* (LC)	When cow refuse its calf, muscle stiffness/ataxia and inappetence	–	Wound and skin infection [104]	–	–	–	–
*Senna didymobotrya* (LC)	BEF, inappetence, bloat and wound	Wound [29], bloat [87]	–	AHS, blackleg, snake bite, ectoparasites and respiratory ailments [33], deworming and Salmonellosis [92]	–	–	–
*Senna occidentalis* (LC)	Snake bite, snake breath, inappetence, colic, diarrhoea, anthrax, bloat and BEF	–	–	Anthrax [36]	–	Anthrax [108]	–
*Senna petersiana* (LC)	Rabies	–	–	–	–	Food poison and leech [108]	–
*Sida schimperiana*	Prophylaxis against rabies	African horse sickness [19], prenatal abortion [91]		Rabies and preventing bitch birth [120], blackleg [16]		Blackleg, dermatophilosis, LSD, FMD and constipation [54], diarrhoea and tryps [108], wound [111], mental problem [112], indigestion and bloat [110]	Bone and joint dislocation [70, 98–100], abortion [98, 99]
*Solanum incanum* (LC)	Coughing, mouth disease, nasal discharge and ringworm	Leech [87, 88], eye infection [27], febrile illness [87], rabies [91]	Blackleg [104, 114], anthrax [114], CCPP [18, 104], pneumonia [18], lung infection [104]	Blackleg [13, 16], ticks and scabies [120], cowdriosis, dermatophilosis, pasteurellosis, skin diseases, blood clotting and colic [13], colic [22], pasteurellosis [93], wound [30, 36], milking phobia [125], anthrax and snake bite [30]	Ticks, infertility, ringworm, swollen joints, pneumonia and mastitis [31]	Epizootic lymphangitis [122], endoparasites/abdominal pain, bovine/ovine pasteurellosis/lung worm [54], cough [124], anthrax, retained placenta and sudden illness [110]	Wound [97, 99], anthrax [99], abdominal pain [99, 100, 115], diarrhoea, shivering and leech [100], swelling, eye disease and LSD [115]
*Marsdenia schimperi*	Mange/alopecia, Blackleg, Anthrax, BEF and LSD	Snake bite and wound [19]	–	Hepatitis and lameness [22]	–	–	Rabies [98, 99]
*Tapinanthus globifer*	Muscle stiffness/ataxia and inappetence	–	–	Anthelmintics [127]		Anthrax, blackleg and sudden illness [110]	–
*Vachellia sieberiana* (LC)	Dullness, Muscle stiffness/ataxia and inappetence	Wound [29]	–	Skin diseases [36]	–	–	–
*Vachellia tortilis* (LC)	Fattening, strengthening and inappetence	–	Brucellosis [104]	Diarrhoea and surgery [17], ectoparasite [92]	Wound [31]	–	–
*Vepris nobilis* (LC)	Anthrax	–	–	Thinness and anthrax [119]	–	–	–
*Verbascum sinaiticum*	Diarrhoea, bloat, mange/alopecia, pasteurellosis, inappetence, scabies, colic and prophylaxis against rabies	Blackleg [27, 28], antipain [29], thinning, rabies, tryps, eye infection and mich [88], gastrointestinal ailments, anthrax, pneumonia, diarrhoea and equine colic [27, 89], rabies [86]	–	Blackleg [32, 120], anthrax [117], loss of appetite [32], skin diseases [33, 107], antipyretic, spleen and liver problem [92], ectoparasite [119], anthelmintics [127]	–	Urinary retention, indigestion and sudden illness [110]	Wound [97, 99], anthrax [98, 99, 103], bone dislocation [99], swelling [100], uroliths [70]
*Verbena officinalis*	Eye disease	Stop bleeding after birth, wound and diarrhoea [116]	–	–	–	Snake bite/poison [122], diarrhoea [111]	Stomachache [113]
*Viscum tuberculatum*	Muscle stiffness/ataxia and inappetence	–	–	AHS [33], poisons, snake venom, shivering and abnormal breathing [118], acute disease [94]	–	–	–
*Withania somnifera*	Diarrhoea, bloat, colic, mange/alopecia, evil eye, blackleg, anthrax, BEF and LSD	Blackleg [19, 88], tania [27]	Listeriosis and blackleg [18]	Stimulate lactation and evil eye [33], evil spirit [16], bloat, antitoxic and appetizer [92], anthelmintics [127], skin diseases [107], anthrax [36]	Urinary abnormalities [31]	Blackleg and endoparasite/abdominal pain [54, 108], diarrhoea [54, 95, 108], anthrax and wound [30], tryps and tsetse fly control [54], swelling, skin problem and snake bite [108], febrile illness [95]	Eye infection [99], bloat [100], swelling, evil spirit and eye disease [115]

CS: Conservation status; LC: Least concern, VU: Vulnerable; DD: Data Deficient; lAmR: Amhara Region; AfR: Afar Region; OR: Oromia Region; SR: Somali Region; SNNPR: Southern Nation, Nationalities and Peoples Region; TR: Tigray Region, BER: Bovine ephemeral fever, FMD: Foot and mouth disease, CCPP: contagious caprine pleuropneumonia; CBPP: contagious bovine pleuropneumonia; PPR: Peste des petits ruminants; LSD: Lumpy skin disease; Tryps: trypanosomiasis; AHS: African horse sickness

Some reported medicinal plants are also widely used as ethnoveterinary medications in different countries of the world too [26, 59–61, 64, 65, 67, 68, 76, 81–85]. Just to mention a few *Allium sativum*, *Melia azedarach*, *Calotropis procera*, *Cissus quadrangularis*, *Foeniculum vulgare*, *Nicotiana tabacum*, *Opuntia ficus-indica* and *Withania somnifera*. This similarity could be due to mixing of cultural knowledge through globalization, substitution of native species for exotic, naturalization of exotic species, cosmopolitan nature of species in use and its associated knowledge [60]. The substitution of native species by exotic ones have tremendous effects on the survival of native species and the knowledge associated with them, which could be lost forever, thus it needs research into documentation of information about neglected native plants and their uses [60]. Perhaps, documentation of traditional knowledge of native or exotic ethnoveterinary medicinal plant species is crucial as they help in providing herbal materials for the discovery of new low-cost drugs that are environmentally friendly and conservation of biodiversity [19].

In this study, eight ethnoveterinary medicinal plants were reported for the first time in treatment of livestock ailments. These medicinal plants include *Capparis fascicularis* for treatment of eye, *Desmidorchis retrospiciens* for constipation, *Dombeya torrida* for mange, diarrhoea and colic. *Erianthemum dregei* and *Loranthella schimperi* for muscle stiffness/ataxia and inappetence, *Oreosyce africana* for anthrax and equine diseases. *Pappea capensis* for treatment of new born rejection (poor mothering) in cattle and *Persicaria decipiens* is used to treat emaciation and remove retained placenta. Thus, these plants need to be studied for their phytochemical constituents and biological activities, which could be important components in future veterinary pharmaceuticals.

Some new ethnoveterinary uses of medicinal plants are also recorded in the study area. For instance, traditional remedy prepared by concoction of four medicinal plants including *Phytolacca dodecandra* (leaves), *Calpurnia aurea* (seed), *Cyphostemma cyphopetalum* (root) and *Verbascum sinaiticum*, then mixed with milk or blood and given to dogs, one drop to their left nostril and ear, which is used for prophylaxis against rabies. In some sites, the remedy also formulated from grounded seeds of *C. aurea* mixed with milk or blood, then given to dogs following the same procedure. According to local people and traditional healers, the remedy is used as traditional vaccine against rabies. The remedy is given to the healthy dog to prevent rabies, which serve at least for one year. As rabies is one of deadly disease in Ethiopia and elsewhere in the world, such medicinal indications are important for its prevention and need further investigations.

### Conservation status, threats to ethnoveterinary plants and indigenous knowledge

The conservation status of reported ethnoveterinary medicinal plants were checked on the International Union for Conservation of Nature (IUCN) Red List. According to the IUCN Red List, 20 (31.25%) reported ethnoveterinary medicinal plants are documented in different conservation status, which includes 18 (28.125%) species that are least concern (LC), 2 species that are vulnerable (VU) and data deficient (DD). The remaining 44 medicinal plants are not documented/classified on the IUCN Red List, this might indicates their conservation status not assessed or they have least threats to conservation.

In the study area, the major threats and challenge to medicinal flora are anthropogenic disturbances and ecological degradation as elsewhere in Ethiopia [77], these include expansion of farmlands, overgrazing, deforestation and soil erosion. Most natural land areas have encroached by inhabitants and converted to agricultural lands mainly into mono-cropping system that led to decline in the multipurpose and medicinal plants in the District. Medicinal plants are also highly affected by overgrazing as large livestock population are freely grazing in *Acacia* woodland, wooded grassland and wetlands, by reducing their regeneration ability. The other important threats are cutting trees for various purposes (firewood, charcoal and construction), urbanization, infrastructure construction and mineral mining. The lack of conservation practices in cultivating of medicinal plants and harvesting from the wild have also led to deterioration of medicinal resources. Similarly, ethnoveterinary knowledge associated with medicinal plants are also being threatened by aforementioned threats coupled with acculturation, weakening of social structures, secrecy, verbal modes of knowledge transfer and difficulty in understanding the knowledge. Nowadays, application of ethnoveterinary knowledge has decreased in the study area due to increase of modern drugs and education, weak interaction of young generation with traditional societies and natural environments. Therefore, to sustain and conserve ethnoveterinary medicinal plants and associated indigenous knowledge, it need revitalization of traditional institution like *Gada* system for biodiversity conservation, which prohibit cutting of respected plants in *Gada* practicing areas (Arda Jila/Malka) and sacred place in the study area. Furthermore, awareness creation among local inhabitants, women and young generation about ethnoveterinary medicinal plant knowledge. And developing medicinal plants utilization strategies, integration into modern livestock health care and promulgation policies in coordination with various stakeholders [21, 77].

## Conclusions

The study revealed that the people of Dugda District are endowed with rich ethnoveterinary knowledge, practices and medicinal plants. As a result, a total of 64 medicinal plants were identified and documented along with their detailed veterinary uses and to be used to treat a wide ranges of livestock ailments. Anthrax, inappetence and diarrhoea were the most prevalent and treat by large number of medicinal plants. The study also showed that ethnoveterinary knowledge of medicinal plants have a remarkable value to deal with these livestock health problems, particularly in areas with limited access to modern pharmaceuticals and rural poor populations. Family Fabaceae was the most important in ethnoveterinary uses (7 spp.), followed by Apocynaceae, Cucurbitaceae and Solanaceae (5 spp. each) and Malvaceae and Vitaceae (4 spp. each). Herbs were the most dominant life forms (21 spp.), followed by shrubs (20 spp.). Leaves (55.25%) were the most sought plant part in ethnoveterinary remedy formulation. The principal method of remedies preparation was pounding remedial parts (46.85%), and mixing with cold water. The main route of administration was via oral application (72.67%), by drenching diseased livestock. *Withania somnifera* and *Kedrostis foetidissima* were the most cited medicinal plants with 53UR and 43UR, respectively*.* ICF showed that respiratory diseases scored the highest value (0.94), while most of the reported medicinal plants were gastrointestinal agents. Multipurpose/use diversity analysis revealed that *Croton macrostachyus* had highest diversity of uses, followed by *K. foetidissima* and *W. somnifera*. Ethnoveterinary uses of some medicinal plants such as *Phytolacca dodecandra*, *Calpurnia aurea*, *Cyphostemma cyphopetalum* and *Verbascum sinaiticum* in rabies prevention were a new input for ethnoveterinary database. Most importantly, majority of medicinal plants were freshly collected from natural habitants (wild); moreover, roots and whole plant parts of some medicinal plants are harvested for ethnoveterinary medications. These in turn have negative implications on conservation of these vitally important ethnoveterinary medicinal plants. Thus, findings of this study could be for conservation of indigenous knowledge and plants diversity. This could be done through implementation of ex situ and in situ conservation actions for medicinal plants, plant biodiversity in general, as these natural resources are being scarce due to several anthropogenic threats, in collaborations with governments, non-governments and traditional institutions (*Gada* system). The agricultural offices at different levels, education sectors and non-governments institutions should encourage indigenous communities to develop senses of ownership and active participation in sustainable management and conservation of medicinal plant biodiversity. Medicinal plants should be promoted for ethnoveterinary services by ethnobotanists, veterinarians and other stakeholders, so that local communities start to conserve these plants and use for management of livestock ailments. Furthermore, the disparity in ethnoveterinary knowledge tied with medicinal plant species (*P* < 0.05) between types of informants, age groups and literacy level also infers the conservation of medicinal plants and associated ethnoveterinary knowledge through awareness creation among local communities and revitalization of the knowledge. The reported medicinal plants could also be potential resources for present and future generations in improving livestock and human health problems, food security, strengthening the livelihoods of local communities and alleviating poverty, which indeed needs conservation and sustainable utilization. Lastly, professionals including ethnobotanists, veterinarians, pharmacologists and biochemists should work together to screen ethnoveterinary medicinal plants for their phytochemical constituents, pharmacological properties and toxicity to confirm ethnoveterinary uses and for future development of veterinary pharmaceuticals.

## Figures and Tables

**Fig. 6:**
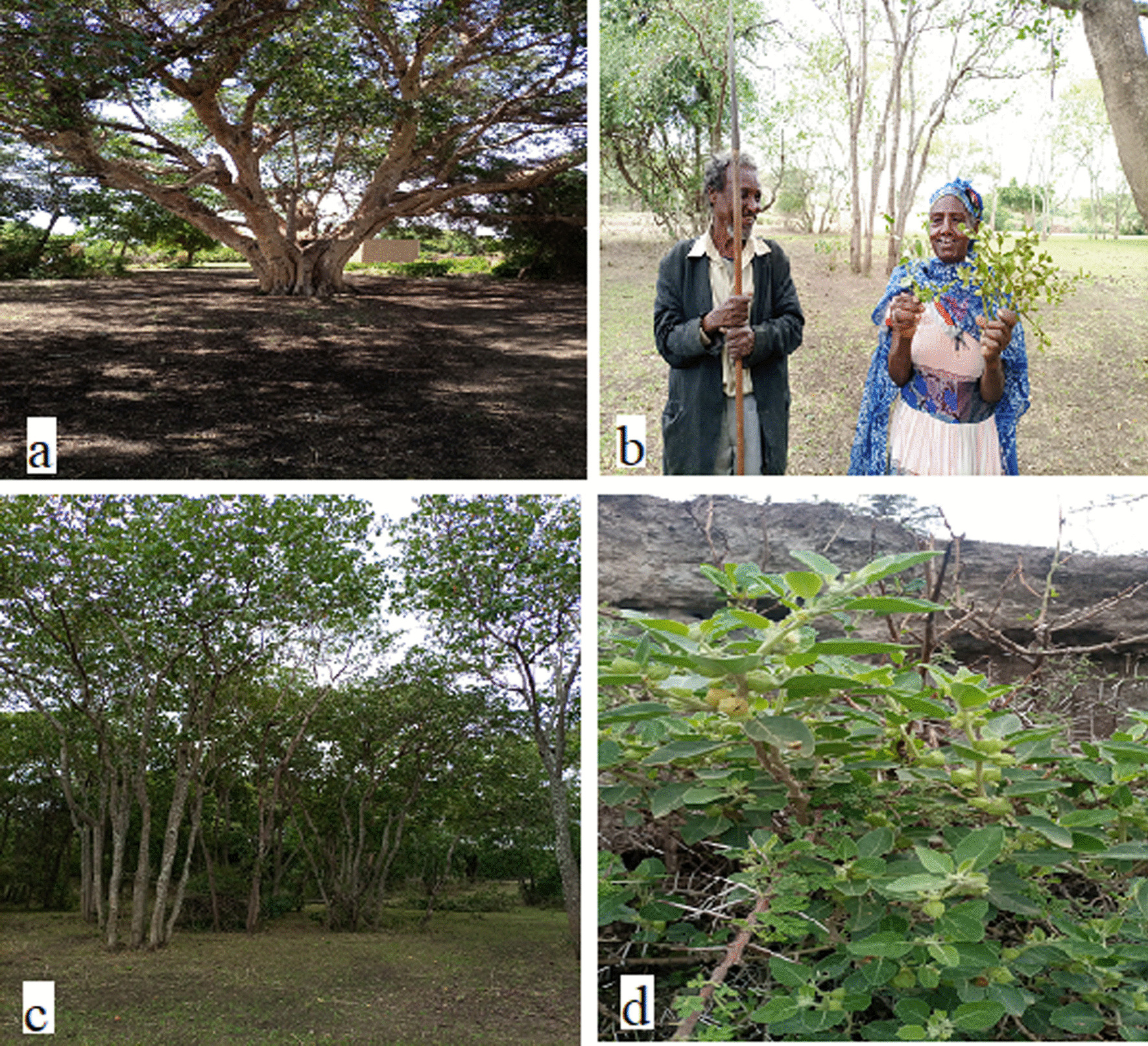
Photograph of sacred tree in the visited study area, informants demonstrating medicinal plant, woodland as sources of medicinal plants and widely used medicinal plant (*Withania somnifera*) respectively

## Data Availability

All the data used to support this study are included in the paper.

## Appendix A1:

See Table 9.

**Table 9:** List of ethnoveterinary medicinal plants in single plant remedy preparations/formulations

Scientific name	Family name	Local name	LF	Habitats	Ailment treated and livestock types	PPU	CPU	MPA	RA	Col. No
*Achyranthes aspera* L	Amaranthaceae	Darguu araba	H	Fe, Fm, Tm	Diarrhoea (c)	Ro	F	Grind, add water and drenching	O	BK 67
					Equine diseases (d, h, m)	Ro	F	Grind, add water and drenching	N	
*Allium sativum* L. **^	Amaryllidaceae	Qullubbii adii	H	Hg	Anthrax (c)	Bu	F	Pound, mix with water and drenching	O	BK 126
*Aloe trichosantha* A.Berger ***	Asphodelaceae	Hargiisa	H	Fm, Te, Hg, Rh	Mange/Alopecia (c)	La	F	Squeeze and drops	De	BK 10
					LSD (c)	La	F	Squeeze and drops	De	
					Wound (c, g, s)	La	F	Squeeze and dropping	De	
					Hump swelling (c)	Le	F	Heat and cauterization	De	
*Asparagus africanus* Lam	Asparagaceae	Sariitii	C	Cf, Fe, PL, Rh	Wound (c, g, s)	Le	D	Grind and pasting	De	BK 05
*Balanites aegyptiaca* (L.) Delile	Zygophyllaceae	Baddannoo/Baddana	T	Cf, WL, PL, Rh	Anthrax (c)	Sb	F	Grind, mix with water and drenching	O	BK 06
					Blackleg (c)	St	F	Heat and cauterization	De	
*Calotropis procera* (Aiton) Dryand	Apocynaceae	Buna gadhee	S	Rs, Rh	Wart (c)	La	F	Squeeze and dropping	De	BK 57
					Ringworm (c)	La	F	Squeeze and dropping	De	
					Blackleg (c)	La	f	Squeeze, mix with butter and smearing	De	
*Calpurnia aurea* (Aiton) Benth	Fabaceae	Ceekaa	S	WL, PL, Ri, Fe	Ticks infestation (c, g, s)	Le	F	Pound, mix with small water and pasting	De	BK 40
					Lice (c, g, s)	Le	F	Pound, mix with small water and pasting	De	
					Prophylaxis against rabies (do, ca)	Se	F/D	Grind, mix with milk/blood and drops in nose and ear	O/N/E	
					Rabies (All livestock)	Le	F	Pound, mix with water and drenching	O	
*Capparis fascicularis* DC	Capparaceae	Goraa baddee	C	Ri, Rh	Eye disease (c, g, s)	Fr	F	Chew and spitting	Op	
*Capparis tomentosa* Lam	Capparaceae	Goraa	C	Tm, Fm, WL, PL, Ri	Fattening and strengthen (c)	Fr	F	Raw and feeding	O	BK 31
*Capsicum frutescens* L. **^	Solanaceae	Mixmixa	H	Hg	When cow refuse its calf (c)	Fr	D	Grind and fumigating	N	
*Cissus quadrangularis* L	Vitaceae	Cophii	C	Fe	Anthrax (c)	Ro	F	Pound, mix with water and drenching	O	BK 117
*Crinum abyssinicum* Hochst. ex A.Rich	Amaryllidaceae	Burii waraabessaa	H	GL, WL, Ri	Rabies (All livestock)	Wp	F	Pound, add water, then drenching and spraying	O/De	BK 121
*Croton macrostachyus* Hochst. ex Delile	Euphorbiaceae	Bakkanniisa	T	GL, WL, Ri, PL	Mange/Alopecia (c)	Ys	F	Pound, add water and drenching	O	BK 27
					Bloat (c)	Le	F	Pound, add water and drenching	O	
					Diarrhoea (c)	Le	F	Pound, add water and drenching	O	
					Inappetence (c)	Ys	F	Pound, add water and drenching	O	
					Bone fracture (All livestock)	Le	F	Decoction and washing	De	
					Dullness (c)	Sb	F	Pound, add water and drenching	O	
*Cucumis ficifolius* A.Rich	Cucurbitaceae	Holotoo gurraattii	C	Fe, GL, FL	Bloat (c)	Ro	F	Grind, mix with water and drenching	O	BK 54
					Blackleg (c)	Ro	F	Grind, mix with water and drenching	O	
					Diarrhoea (c)	Ro	F	Grind, mix with water and drenching	O	
					Mineral deficiency (c)	Ro	F	Grind, mix with water and drenching	O	
					Mange/Alopecia (c)	Ro	F	Grind, mix with water and drenching	O	
*Cynoglossum lanceolatum* Forssk	Boraginaceae	Maxxannee xafaa	H	Cf, GL	Inappetence (c)	Le	F	Pound, add water and drenching	O	BK 21
*Cyphostemma adenocaule* (Steud. ex A. Rich.) Desc. ex Wild & R.B.Drumm	Vitaceae	Midhaan qochaa	C	Fe	BEF (c)	Ro	F	Grind, mix with water, salt and skimmed milk, then drenching	O	BK 29
					Inappetence (c)	Ro	F	Grind, mix with water, salt and skimmed milk/whey, then drenching	O	
					Anthrax (c)	Ro	F	Grind, mix with water and drenching	O	
					Lymphangitis (h)	Ro	F	Grind, mix with kerosene and butter/ghee and pasting	De	
*Cyphostemma cyphopetalum* (Fresen.) Desc. ex Wild & R.B.Drumm	Vitaceae	Hindirifaa	C	Fe	Anthrax (c)	Ro	F	Decoction and drenching	O	BK 53
					Bloat (c)	Ro	F	Pound, add water and drenching	O	
					Colic (c)	Ro	F	Pound, add water and drenching	O	
					Prophylaxis against rabies (do, ca)	Ro	F	Grind, add milk/blood, drinking, nose and eardrops	O/N/E	
					Diarrhoea (c)	Ro	F	Pound, add water and drenching	O	
*Cyphostemma pannosum* Vollesen	Vitaceae	Cophii	H	WL, Ri	BEF (c)	Le/R	F	Pound, add water and drenching	O	BK 180
					Equine diseases (d, h, m)	Le/R	F	Pound, add water and drenching	O	
					Mange/Alopecia (c)	Le/R	F	Pound, add water and drenching	O	
*Datura stramonium* L. **	Solanaceae	Manjii	H	Cf, GL, FL	Rabies (All livestock)	Le/Ro	F	Decoction and drenching	O	BK 149
					Wound (c, g, s)	Le	F	Pound, mix with small water and pasting	O	
*Desmidorchis retrospiciens* Ehrenb	Apocynaceae	Adaamii dooluu/ooluu	H	Rh	Constipation (c)	Le	F	Pound, mix with water and drenching	O	BK 12
*Dodonaea viscosa* subsp. *angustifolia* (L.f.) J.G.West ***	Sapindaceae	Ittacha	S	Hs, Rh	Mange/Alopecia (c)	Le	F	Pound, mix with water and drenching	O	BK 38
*Dombeya torrida* (J. F. Gmel.) P. Bamps	Malvaceae	Daannisaa	T	PL, WL	Mange/Alopecia (c)	Le	F	Decoction and drenching	O	BK 62
					Diarrhoea (c)	Le	F	Decoction and drenching	O	
					Colic (c)	Le	F	Pound, mix with water and drenching	O	
					Colic in equine (d, h, m)	Le	F	Pound, mix with water and drenching	O	
*Echinops hispidus* Fresen	Asteraceae	Sookorruu	H	Fe	Inappetence (c)	Wp	F	Pound, add water and drenching	O	BK 55
*Foeniculum vulgare* Mill	Apiaceae	Insilaalee	H	Cf, Fe, Hg	Mange/Alopecia (c)	Ro	F	Grind, add water and drenching	O	BK 59
					Urine retention/urolithiasis (c)	Ro	F	Grind, add water and drenching	O	
*Gomphocarpus fruticosus* (L.) W.T.Aiton	Apocynaceae	Damma saree/Hanxiffachiisaa	S	Cf, GL, Fm, Rs	Ringworm (c)	Le	D	Grind, mix with butter and pasting	De	BK 183
					Foot and Mouth Disease (c)	Wp	D	Grind, mix with water, then drenching and pasting	O/De	
					Scabies (c, g, s)	Le	D	Grind, mix with butter and pasting	De	
					Wart (c)	Ro	D	Grind, mix with butter and pasting	De	
*Gossypium hirsutum* L. **^	Malvaceae	Jirbii	S	Fe, Hs	Eye disease (c, g, s)	Se	D	Chew and spitting	Op	
*Grewia ferruginea* Hochst. ex A.Rich	Malvaceae	Dhoqonuu	S	Ri	Retained placenta (c, g, s)	Sb	F/D	Soak in water and drenching	O	BK 37
*Gymnanthemum amygdalinum* (Delile) Sch.Bip	Asteraceae	Ebicha	S	Hs, Ri, Cf	Evil spirit (c)	Le	F	Pound, mix water and spraying	De	BK 49
					Diarrhoea (c)	Le	F	Pound, add water and drenching	O	
*Ipomoea carnea* Jacq. **	Convolvulaceae	Saara	C	Fe	Rabies (All livestock)	Wp	F/D	Pound, mix with water and drenching	O	BK 107
*Kalanchoe petitiana* A.Rich. *	Crassulaceae	Luqqee	H	Fe, Rh, GL	Hump swelling (c)	Ro	F	Slice, cut dewlap and insert	De	BK 07
*Kedrostis foetidissima* Cogn	Cucurbitaceae	Manaabaasii/Tirinyii	C	Fe	Diarrhoea (c, g, s)	Le	F	Pound, mix water and salt, then drenching	O	BK 113
					Bloat (c, g, s)	Le	F	Pound, mix water and salt, then drenching	O	
					Fattening and strengthening (c)	Le	F	Pound, mix water and salt, then drenching	O	
					BEF (c)	Le	F	Pound, mix water and salt, then drenching	O	
					Lice (c, g, s)	Le	F	Pound, mix with small water and pasting	De	
					Retained placenta (c, g, s)	Le	F	Pound, mix with water and drenching	O	
					Lymphangitis (h)	Le	F	Pound, mix with water and drenching	O	
*Linum usitatissimum* L. **^	Linaceae	Talbaa	H	Cf	Retained placenta (c)	Se	D	Grind, add water and drenching	O	BK 182
*Melia azedarach* L. **^	Meliaceae	Miimmii	T	Hs	Colic (c)	Le	F	Pound, mix with water and drenching	O	BK 124
					Bloat (c, g, s)	Le	F	Pound, mix with water and drenching	O	
					Diarrhoea (c, g, s)	Le	F	Pound, mix with water and drenching	O	
					Inappetence (c)	Le	F	Pound, mix with water and drenching	O	
					Chicken diseases	Le	F	Pound, mix with water and drinking	O	
*Momordica foetida* Schumach	Cucurbitaceae	Gaallee quxusuu	C	Fe	Bloat (c)	Le	F	Pound, adding water and drenching	O	BK 114
*Nicotiana tabacum* L. **^	Solanaceae	Tamboo	H	Hg	Coughing (c, g, s)	Le	D	Grind, mix with small water and dropping	N	BK 148
					Nasal discharge (c, g, s)	Le	D	Grind, mix with small water and dropping	N	
*Opuntia ficus-indica* (L.) Mill. **	Cactaceae	Adaamii	T	Fm, Fe, Gm	Emaciation (c)	Le	F	Pound, mix with water and drenching	O	BK 158
					Diarrhoea (c)	Le	F	Pound, mix with water and drenching	O	
*Oreosyce africana* Hook.f	Cucurbitaceae	Dimmiigaa/Midhaan bofa	C	WL, PL, Rh	Anthrax (c)	Tu	F	Chop, grind, adding water and drenching	O	BK 66
					AHS (h)	Tu	F	Chop, grind, adding water and drenching	O	
*Pappea capensis* Eckl. & Zeyh	Sapindaceae	Biiqqaa	T	Cf	When cow refuse its calf (c)	Le	F	Chew and spitting	Su	BK 184
*Persicaria decipiens* (R. Br.) K.L.Wilson	Polygonaceae	Haxaawwii	H	We, Da	Emaciation (c)	Le	F	Pound, mix with water and drenching	O	BK 181
					Retained placenta (c, g, s)	Le	F	Pound, mix with water and drenching	O	
*Phytolacca dodecandra* L'Her	Phytolaccaceae	Andoodee	C	Fe, Fm, Ri	Ringworm (c)	Le	F	Pound, mix with water and washing	De	BK 11
					Rabies (All livestock)	Ro	F	Crush, mix with small water and food, then drenching	O	
					Submandibular oedema (c, g, s)	Le	F	Pound, mix with water and drenching	O	
					Eye disease (c, g, s)	Le	F	Pound, mix with water and washing	Op	
					Foot and Mouth Disease (c)	Le	F	Pound, mix with water and drenching	O	
					BEF (c)	Le	F	Pound, mix with water and drenching	O	
					Constipation (c)	Le	F	Pound, mix with water and drenching	O	
*Pterolobium stellatum* (Forssk.) Brenan	Fabaceae	Hallanqabeessa/Harangamaa	S	Ri, Rh	Evil eye (c)	Ro	F/D	Grind, add water, then drenching, burning and fumigation	O/N	BK 119
*Searsia natalensis* (Bernh. Ex C.Krauss) F.A.Barkley	Anacardiaceae	Daboobessa	S	PL, WL, Fm, Fe, Tm	When cow refuse its calf (c)	Le	F	Chew and suppositories	Su	BK 36
*Senna didymobotrya* (Fresen.) H.S.Irwin & Barneby ***	Fabaceae	Birbirraa	S	Fe, Fm	BEF (c)	Le	F	Pound, mix with water, salt and kerosene, then drenching	O	BK 139
					Inappetence (c)	Le	F	Pound, mix with water and drenching	O	
					Bloat (c)	Le	F	Pound, mix with water and drenching	De	
					Wound (c, g, s)	Le	F	Pound, mix with water and drenching	De	
*Senna occidentalis* (L.) Link **	Fabaceae	Shiwwaashiwwee/Baala bofaa	H	Fm, GL	Snake bite (All livestock)	Le	F	Pound, mix with water and drenching	O	BK 48
					Snake breath (All livestock)	Le	F	Decoction and washing	De	
					Inappetence (c)	Le	F	Pound, mix with water and drenching	O	
					Colic (c)	Le	F	Pound, mix with water and drenching	O	
					Diarrhoea (c)	Le	F	Pound, mix with water and drenching	O	
*Senna petersiana* (Bolle) Lock	Fabaceae	Birbirraa	S	Cf, Rh	Rabies (All livestock)	Le	F	Pound, mix with water and drenching	O	BK 108
*Solanum incanum* L	Solanaceae	Hiddii	S	WL, PL, GL	Coughing (g, s)	Fr	F	Squeeze and dropping	N	BK 45
					Mouth disease (g, s)	Fr	F	Squeeze and pasting	De	
					Nasal discharge (g, s)	Fr	F	Squeeze and dropping	N	
					Ringworm (c)	Fr	F	Squeeze and dropping	N	
*Marsdenia schimperi* Decne	Apocynaceae	Qaraanqabdoo	C	Rh, Fm, WL, Fe	Mange/Alopecia (c)	Le	F	Pound, mix with water, then drenching and pasting	O/De	BK 03
*Vachellia sieberiana* (DC.) Kyal. & Boatwr	Fabaceae	Fullisa	T	Cf, PL, WL	Dullness (c)	Le	F	Pound, mixing with water and drenching	O	BK 35
*Vachellia tortilis* (Frossk.) Gallaso & Banfi	Fabaceae	Addacha	T	Cf, PL, WL	Fattening and strengthening (c)	Fr	F/D	Raw and feeding	O	BK 41
					Inappetence (c)	Le	F	Pound, with water and drenching	O	
*Vepris nobilis* (Dellile) Mziray	Rutaceae	Hadheessa	T	Cf, PL, WL	Anthrax (c)	Le	D	Grind, mix with water and dropping	N	BK 64
*Verbascum sinaiticum* Benth	Scrophulariaceae	Gurra harree	H	Rh	Diarrhoea (c)	Ro	F	Grind, add water and drenching	O	BK 60
					Bloat (c)	Le	F	Pound, mix with water and drenching	O	
					Mange/Alopecia (c)	Ro	F	Pound, mix with water and drenching	O	
					Pasteurellosis (c)	Le/Ro	F	Pound, mix with water and drenching	O	
					Inappetence (c)	Le/Ro	F	Pound, mix with water and drenching	O	
					Scabies (c, g, s)	Le	F	Pound, mix with small water and pasting	De	
					Colic (c)	Le	F	Pound, mix with water and drenching	O	
*Verbena officinalis* L	Verbenaceae	Qoricha Ijaa	H	Cf, Fm	Eye disease (c, g, s)	Le	F	Crush, mix with water and salt and dropping/spitting	Op	BK 90
*Withania somnifera* (L.) Dunal	Solanaceae	Waahallee/Daadhoo	S	Fm, Tm, WL, Hg, Rh	Diarrhoea (c)	Le	F	Pound, mix with water and drenching	O	BK 01
					Evil eye (c)	Ro	F/D	Grind and tie on neck and fumigation	De/N	
*Xanthium spinosum L. ***	Asteraceae	Arraba dubartii	H	Cf	Inappetence (c)	Wp	F	Pound, add water and drenching	O	BK 162
*Ziziphus mucronata* Willd	Rhamnaceae	Qurquraa	T	Cf, PL, WL	Diarrhoea (c)	Le	F	Pound, add water and drenching	O	BK 25
					Mange/Alopecia (c)	Le	F	Pound, add water and washing	De	

* = Endemic, ** = Exotic, *** = Semi-wild, **^ = Exotic and cultivated, LF = Life form (C = Climber, H = Herb, S = shrub, T = Tree), Habitats (Fe = Fences, Fm = Field margins, Tm = Termite mounds, Hg = Homegardens, Te = Terracing, Rh = Rocky hills, Cf = Crop fields, PL = Parklands, WL = Woodlands, Rs = Road sides, Ri = Riverines, GL = Grazing lands, Hs = Homesteads, Gm = Grave marker, We = Wetlands, Da = Damp areas, FL = Fallow lands, Hp = Host plants, Sa = Shade areas), Livestock types (c = cattle, g = goats, s = sheep, d = donkeys, h = horses, m = mules, do = dogs, ca = cats), PPU = plant part used (Bu = Bulb, Fr = Fruit, La = Latex, Le/Ro = Leaves/Root, Le = Leaves, R = Root, St = Stem, Sb = Stem bark, Tu = Tuber, Wp = Whole part, Ys = Young shoot), CPU = Condition of parts used (D = Dry, F = Fresh, F/D = Fresh/Dry), MPA = Methods of Preparation and Application, RA = Routes of Administration (De = Dermal, De/N = Dermal/Nasal, N = Nasal, Op = Optical, O = Oral, O/De = Oral/Dermal, O/N = Oral/Nasal, O/N/E, Su = Suppositories

## Appendix A2

See Table 10.

**Table 10:** List of ethnoveterinary medicinal plants in poly-herbal remedy preparations/formulations

Scientific name	Family name	Local name	LF	Habitats	Ailment treated and livestock types	PU	CPU	MPA	RA	Col. No
*Withania somnifera* (L.) Dunal	Solanaceae	Waahallee/Daadhoo	S	Fm, Tm, WL, Hg, Rh	Bloat (c), Colic (c), Mange/Alopecia (c)	Le	F	Pound, mix with water and salt, then drenching, drenching and pasting (in case of mange treatment)	O	BK 01
*Gymnanthemum amygdalinum* (Delile) Sch.Bip	Asteraceae	Ebicha	S	Hs, Ri, Cf		Le			O/De	BK 49
*Asparagus africanus* Lam	Asparagaceae	Sariitii	C	Cf, Fe, PL, Rh	Rabies (All livestock)	Le	F	Pound, mix with water and milk, then drenching	O	BK 05
*Datura stramonium* L	Solanaceae	Manjii	H	Cf, GL, FL						BK 149
*Phytolacca dodecandra* L’Her	Phytolaccaceae	Andoodee	C	Fe, Fm, Ri	Prophylaxis against rabies (do, ca)	Le	F	Pound, mix with milk/blood, then drinking and drops in nose and ear	O/N/E	BK 11
*Calpurnia aurea* (Aiton) Benth	Fabaceae	Ceekaa	S	WL, PL, Ri, Fe		Se	F/D			BK 40
*Cyphostemma cyphopetalum* (Fresen.) Desc. ex Wild & R.B.Drummond	Vitaceae	Hindirifaa	C	Fe		Ro	F/D			BK 53
*Sida schimperiana* Hochst. ex A.Rich	Malvaceae	Guftee	S	Fm, WL, PL, GL	Prophylaxis against rabies (do, ca)	Le	F	Pound, mix with milk/blood, then drinking and drops in nose and ear		BK 73
*Calpurnia aurea* (Aiton) Benth	Fabaceae	Ceekaa	S	WL, PL, Ri, Fe		Se	F/D			BK 40
*Verbascum sinaiticum* Benth	Scrophulariaceae	Gurra harree	H	Rh		Ro	F			Bk 60
*Cyphostemma cyphopetalum* (Fresen.) Desc. ex Wild & R.B.Drummond	Vitaceae	Hindirifaa	C	Fe	Blackleg (c), Anthrax (c), BEF (c), LSD (c)	Ro	F	Pound, mix with water and salt, then drenching	O	BK 01
*Withania somnifera* (L.) Dunal	Solanaceae	Waahallee/Daadhoo	S	Fm, Tm, WL, Hg, Rh		Ro				BK 53
*Dodonaea viscosa* Subsp. *angustifolia* (L. f.) J.G.West ***	Sapindaceae	Ittacha	S	Hs, Rh		Le				BK 38
*Cucumis ficifolius* A.Rich	Cucurbitaceae	Holotoo gurraattii	C	Fe, GL, FL		Ro				BK 54
*Foeniculum vulgare* Mill	Apiaceae	Insilaalee	H	Cf, Fe, Hg		Ro				BK 59
*Gymnanthemum amygdalinum* (Delile) Sch.Bip	Asteraceae	Ebicha	S	Hs, Ri, Cf		Le				BK 49
*Croton macrostachyus* Hochst. ex Delile	Euphorbiaceae	Bakkanniisa	T	GL, WL, Ri, PL		Ys				BK 27
*Marsdenia schimperi* Decne	Apocynaceae	Qaraanqabdoo	C	Rh, Fm, WL, Fe		Le				BK 03
*Cyphostemma cyphopetalum* (Fresen.) Desc. ex Wild & R.B.Drummond	Vitaceae	Hindirifaa	C	Fe	Rabies (All livestock)	Le	F	Pound, mix with water and drenching	O	BK 53
*Lagenaria siceraria* (Molina) Standl. ***	Cucurbitaceae	Buqqee	H	Fe, Cf, Hg		Le/Fr	F			BK 143
*Kedrostis foetidissima* Cogn	Cucurbitaceae	Manaabaasii	C	Fe	Mange/Alopecia (c)	Le	F	Pound, mix with water, then drenching and pasting	O/D	BK 113
*Verbascum sinaiticum* Benth	Scrophulariaceae	Gurra harree	H	Rh		Ro				BK 60
*Achyranthes aspera* L	Amaranthaceae	Darguu arbaa	H	Fe		Ro				BK 67
*Withania somnifera* (L.) Dunal	Solanaceae	Waahallee/Daadhoo	S	Fm, Tm, WL, Hg, Rh		Le				BK 01
*Cyphestemma pannosum* Vollesen	Vitaceae	Cophii	H	Fe		R				BK 180
*Oreosyce africana* Hook.f	Cucurbitaceae	Dimmiigaa/Midhaan bofaa	C	WL, PL, Rh		Tu				BK 66
*Withania somnifera* (L.) Dunal	Solanaceae	Waahallee/Daadhoo	S	Fm, Tm, WL, Hg, Rh	Blackleg (c)	Ro	F	Pound, mix with water and drenching	O	BK 01
*Achyranthes aspera* L	Amaranthaceae	Darguu arbaa	H	Fe, Fm, Tm		Ro				BK 67
*Balanites aegyptiaca* (L.) Delile	Zygophyllaceae	Baddannoo/Baddana	T	Cf, WL, PL, Rh		Sb				BK 06
*Senna occidentalis* (L.) Link	Fabaceae	Shiwwaashiwwee/Baala bofaa	H	Fm, GL	Anthrax (c)	Le	F	Pound, mix with water and salt, then drenching	O	BK 48
*Cucumis ficifolius* A.Rich	Cucurbitaceae	Holotoo gurraattii	C	Fe, GL, FL		Ro				BK 54
*Ocimum gratissimum* subsp. *gratissimum*	Lamiaceae	Haraamuu/Hancabbii	S	Fm, WL, PL, Hg		Ro				BK 08
*Croton macrostachyus* Hochst. ex Delile	Euphorbiaceae	Bakkanniisa	T	GL, WL, Ri, PL	Many disease conditions (c)	Ro	F	Pound, mix with water and drenching	O	BK 27
*Lagenaria siceraria* (Molina) Standl. ***	Cucurbitaceae	Buqqee	H	Fe, Cf, Hg		Ro				BK 143
*Viscum tuberculatum* A.Rich	Santalaceae	Dheertuu Bakkanniisaa	S	Hp	Muscle stiffness/Ataxia (c), Inappetence (c)	Wp	F	Pound, mix with water and drenching	O	BK 02
*Ziziphus mucronata* Willd	Rhamnaceae	Qurquraa	T	Cf, PL, WL		Le				BK 25
*Vachellia sieberiana* (DC.) Kyal. & Boatwr	Fabaceae	Fullisa	T	Cf, PL, WL		Le				BK 34
*Searsia natalensis* (Bernh. Ex C.Krauss) FA.Barkley	Anacardiaceae	Daboobessa	S	PL, WL, Fm, Fe		Le				BK 35
*Loranthella schimperi* (Tiegn.) S.Blanco & C.E.Wetzel	Loranthaceae	Dheertuu Baddannoo	S	Hp		Wp				BK 58
*Tapinanthus globifer* (A. Rich.) Tiegh	Loranthaceae	Dheertuu Daboobessaa	S	Hp		Wp				BK 81
*Erianthemum dregei* (Eckl. & Zeyh.) Tiegh	Loranthaceae	Dhertuu Dhaddachaa	S	Hp		Wp				BK 87
*Gymnanthemum amygdalinum* (Delile) Sch.Bip	Asteraceae	Ebicha	S	Hs, Ri, Cf	Mange/Alopecia (c)	Le	F	Pound, mix with water drenching and pasting	O/De	BK 49
*Opuntia ficus-indica* (L.) Mill. **^	Cactaceae	Adaamii	T	Fm, Fe, Gm		Le				BK 158
*Allium sativum* L. **^	Amaryllidaceae	Qullubbii adii	H	Hg	Anthrax (c), Blackleg (c)	Bu	F	Pound, mix with water and drenching	O	BK 126
*Cyphostemma adenocaule* (Steud. ex A. Rich.) Desc. ex Wild & R.B.Drumm	Vitaceae	Midhaan qochaa	C	Fe		Le				BK 29
*Withania somnifera* (L.) Dunal	Solanaceae	Waahallee/Daadhoo	S	Fm, Tm, WL, Hg, Rh	Bloat (c), BEF (c), Many disease conditions (c)	Ro	F	Pound, mix with water and salt, then drenching	O	BK 01
*Senna occidentalis* (L.) Link **	Fabaceae	Shiwwaashiwwee/Baala bofaa	H	Fm, GL		Le				BK 48
*Senna occidentalis* (L.) Link **	Fabaceae	Shiwwaashiwwee/Baala bofaa	H	Fm, GL	Colic in equines (d, h, m)	Le	F	Pound, mix with water and drenching	N	BK 48
*Hypoestes forskaolii* (Vahl) R.Br	Acanthaceae	Darguu	H	Fm, Sa, Rh, GL, Fe		Le				BK 70
*Kedrostis foetidissima* Cogn	Cucurbitaceae	Manaabaasii	H	Fe		Le				BK 113
*Melia azedarach* L. **^	Meliaceae	Miimmii	H	Hs		Le				BK 124
*Cyphestemma pannosum* Vollesen	Vitaceae	Cophii	H	Fe		Le/R				BK 180
*Phytolacca dodecandra* L’Her	Phytolaccaceae	Andoodee	H	Fe, Fm, Ri	Rabies (All, animals)	Ro	F	Crush, mix with water, then drenching and spraying	O	BK 11
*Carissa spinarum* L	Apocynaceae	Agamsa	S	Fm, PL, WL		Ro				BK 33
*Phytolacca dodecandra* L’Her	Phytolaccaceae	Andoodee	C	Fe, Fm, Ri	Rabies (All animals), Anthrax (c)	Le	F	Pound, mixing with water and drenching	O	BK 11
*Calpurnia aurea* (Aiton) Benth	Fabaceae	Ceekaa	C	WL, PL, Ri, Fe		Le				BK 40
*Withania somnifera* (L.) Dunal	Solanaceae	Waahallee/Daadhoo	C	Fm, Tm, WL, Hg, Rh		Ro				BK 01

* = Endemic, ** = Exotic, *** = Semi-wild, **^ = Exotic and cultivated, LF = Life form (C = Climber, H = Herb, S = shrub, T = Tree), Habitats (Fe = Fences, Fm = Field margins, Tm = Termite mounds, Hg = Homegardens, Te = Terracing, Rh = Rocky hills, Cf = Crop fields, PL = Parklands, WL = Woodlands, Rs = Road sides, Ri = Riverines, GL = Grazing lands, Hs = Homesteads, Gm = Grave marker, We = Wetlands, Da = Damp areas, FL = Fallow lands, Hp = Host plants, Sa = Shade areas), Livestock types (c = cattle, g = goats, s = sheep, d = donkeys, h = horses, m = mules, dg = dogs, ca = cats), PPU = plant part used (Bu = Bulb, Fr = Fruit, La = Latex, Le/Ro = Leaves/Root, Le = Leaves, R = Root, St = Stem, Sb = Stem bark, Tu = Tuber, Wp = Whole part, Ys = Young shoot), CPU = Condition of parts used (D = Dry, F = Fresh, F/D = Fresh/Dry), MPA = Methods of Preparation and Application, RA = Routes of Administration (De = Dermal, De/N = Dermal/Nasal, N = Nasal, Op = Optical, O = Oral, O/De = Oral/Dermal, O/N = Oral/Nasal, O/N/E, Su = Suppositories)

## Appendix A3

See Fig. 6.

## Abbreviations

GDP: Gross domestic product
ACB/RV: *Acacia-Commiphora* Woodland and bushland land/Rift Valley
AAU: Addis Ababa University
ETH: National herbarium
WFO: World flora online
ICF: Informant consensus factor
URs: Use reports
FL: Fidelity-level index
RI: Relative importance values
LSD: Lumpy skin disease
FMD: Foot and mouth disease
CBPP: Contagious bovine pleuropneumonia
BEF: Bovine ephemeral fever
ASH: African horse sickness
IUCN: International Union for Conservation of Nature
